# Cell surface detection of vimentin, ACE2 and SARS-CoV-2 Spike proteins reveals selective colocalization at primary cilia

**DOI:** 10.1038/s41598-022-11248-y

**Published:** 2022-04-29

**Authors:** Vasiliki Lalioti, Silvia González-Sanz, Irene Lois-Bermejo, Patricia González-Jiménez, Álvaro Viedma-Poyatos, Andrea Merino, María A. Pajares, Dolores Pérez-Sala

**Affiliations:** Department of Structural and Chemical Biology, Centro de Investigaciones Biológicas Margarita Salas, CSIC, Ramiro de Maeztu, 9, 28040 Madrid, Spain

**Keywords:** Cellular imaging, Cytoskeleton, Mechanisms of disease, Diseases, Pathogenesis, Proteins, Imaging, Microscopy

## Abstract

The SARS-CoV-2 Spike protein mediates docking of the virus onto cells prior to viral invasion. Several cellular receptors facilitate SARS-CoV-2 Spike docking at the cell surface, of which ACE2 plays a key role in many cell types. The intermediate filament protein vimentin has been reported to be present at the surface of certain cells and act as a co-receptor for several viruses; furthermore, its potential involvement in interactions with Spike proteins has been proposed. Nevertheless, the potential colocalization of vimentin with Spike and its receptors on the cell surface has not been explored. Here we have assessed the binding of Spike protein constructs to several cell types. Incubation of cells with tagged Spike S or Spike S1 subunit led to discrete dotted patterns at the cell surface, which consistently colocalized with endogenous ACE2, but sparsely with a lipid raft marker. Vimentin immunoreactivity mostly appeared as spots or patches unevenly distributed at the surface of diverse cell types. Of note, vimentin could also be detected in extracellular particles and in the cytoplasm underlying areas of compromised plasma membrane. Interestingly, although overall colocalization of vimentin-positive spots with ACE2 or Spike was moderate, a selective enrichment of the three proteins was detected at elongated structures, positive for acetylated tubulin and ARL13B. These structures, consistent with primary cilia, concentrated Spike binding at the top of the cells. Our results suggest that a vimentin-Spike interaction could occur at selective locations of the cell surface, including ciliated structures, which can act as platforms for SARS-CoV-2 docking.

## Introduction

Vimentin is an intermediate filament cytoskeletal protein highly abundant in mesenchymal cells, which is also present in several other cell types, including endothelial cells, certain cells lining the airways and several cells of the immune system, under normal circumstances (https://www.proteinatlas.org/ENSG00000026025-VIM). Moreover, vimentin expression can be increased or induced in cell injury, senescence or during the epithelial-mesenchymal transition that occurs in tumorigenesis or chronic inflammation^1–3^. Vimentin plays important mechanical functions in cells providing support, sustaining the position and protection of cellular organelles, including the nucleus^4–7^, and interacting with the other cytoskeletal systems to contribute to essential cellular functions such as cell division and migration^8–10^. In addition to its functions in the intracellular environment, vimentin has been reported to exert important actions at the cell surface or in the extracellular medium (reviewed in^11,12^). Vimentin has been detected at the surface of diverse cell types, where it acts as a receptor for several types of ligands, such as soluble CD44^13^ or certain carbohydrate chains^14^. Moreover, vimentin itself has been described to be a ligand for various receptors, including IGF-1R^15^ and P-selectin^16^. Circulating forms of vimentin, either in extracellular vesicles or in other non-completely characterized forms, have also been identified^17–19^, which could be involved in autoimmunity, epithelial-mesenchymal transition or in the modulation of inflammatory responses. Importantly, extracellular and/or cell surface vimentin species have been reported to act as co-receptors for several pathogens, including bacteria and viruses, either facilitating or acting as a restriction factor for cellular invasion^20,21^ (reviewed in^12,22^). In the context of viral invasion, vimentin was reported to act as a co-receptor for SARS-CoV, the coronavirus causing the 2003 outbreak, in association with the protein ACE2^23^. The need for therapeutic tools in the fight against SARS-CoV-2, the virus causing the COVID-19 pandemic, has fostered the interest in vimentin as a potential therapeutic target in viral infections, a subject addressed in several recent reviews and hypotheses^12,24,25^.

Research on SARS-CoV-2 represents one of the most extraordinary efforts in Biomedicine, and an enormous amount of resources have been devoted to understand and combat this new pathogen in a record time. The study of SARS-CoV-2 entails high complexity due to the variety of syndromes and symptoms it can provoke. Initially thought to produce a flu-like disease, it is now clear that this virus can invade multiple cell types in the organism and trigger, directly or indirectly, dysfunction of almost any system, and provoke the severe cytokine storm that contributes to the mortality of the disease^26,27^. In an in vitro assay platform SARS-CoV-2 was able to enter cardiomyocytes, pancreatic beta cells, liver organoids and dopaminergic neurons^28^. Indeed, human small intestine organoids are also infected by the virus and support viral replication^29^.

Spike proteins are glycoproteins present at the outer layer of the virus that mediate binding of the viral particles to cellular receptors and membrane fusion. The best characterized receptor for SARS-CoV-2 is ACE2, and detailed structural information on the Spike-ACE2 interaction is available^30,31^. Nevertheless, the ability of this virus to enter diverse cell types^28^ could be related to its capacity to interact with other cellular receptors, which can include integrins^32^, neuropilin^33^, and the tyrosine-protein kinase receptor UFO (AXL)^34^. In addition, membrane-bound lectins, heparan sulfates or specific lipid domains could favor the interaction of the virus with the cell surface^35–38^. The Spike protein possesses two domains which are generated by posttranslational furin cleavage, but remain associated thereafter. The S1 domain contains the receptor binding domain (RBD) that is involved in the interaction with ACE2, whereas the S2 domain is implicated in membrane fusion^31^. Interestingly, both, viral particles and recombinant Spike chimeric proteins containing either the full length protein or specific domains, i.e., S1 or RBD, have been used to explore the interaction with cellular receptors in cell-based assays, as well as in vitro^39,40^. Here, we have carried out the detection of several recombinant versions of the Spike protein, together with endogenous vimentin and/or ACE2, at the surface of different cell types. Our observations highlight the complexity of vimentin immunodetection at the cell surface. Moreover, a highly consistent colocalization of Spike with endogenous ACE2, but lower ACE2-vimentin and Spike-vimentin coincidence were observed. Nevertheless, the three proteins appear to concur at certain cellular structures, in particular, primary cilia, the importance of which in Spike docking deserves further investigation.

## Methods

### Recombinant proteins

SARS-CoV-2 Spike protein constructs, Spike S1-Fc-Avi and Spike S1-His-Avi (Spike protein residues Gln14 to Arg683), Spike S-Fc-Avi (S1 + S2; amino acids Gln14 to Trp1212), and human ACE2-His-Avi (residues Gln18 to Ser740), all expressed in HEK293 cells, were from Bioss Antibodies. Human IgG1-Fc protein “103Cys/Ser” and Cholera toxin subunit B-Alexa555 (CTXB) were from Sino Biological and Molecular Probes, respectively.

### Antibodies

Anti-vimentin antibodies used were: anti-vimentin antibody V9 (sc-6260, unconjugated and Alexa-488-conjugate) from Santa Cruz Biotechnology; mouse monoclonal anti-vimentin clone 13.2 (V5255) from Sigma; rabbit monoclonal SP20 anti-vimentin antibody from ThermoFisher Scientific; cell-surface vimentin (CSV) antibody, clone 84-1, from Abnova, and goat anti-vimentin antibody EB11207 from Everest Biotech. Anti-ARL13B C-5 (sc-515784) was from Santa Cruz Biotechnology, anti-ACE2 rabbit antibodies were purchased from Invitrogen and Abcam, anti-human IgG-Alexa647 and anti-human IgG-Alexa568 were obtained from Invitrogen, anti-acetylated tubulin (T7451) was from Sigma and anti-SARS-CoV-2 Spike protein was a product of Sino Biological. Details of the antibodies used are summarized in Supplementary Table 1.

### Cell culture

Cell culture media and supplements were from Gibco. Fetal bovine serum (FBS) was from Sigma. Fibroblast-like African green monkey kidney cells (Vero) were from the collection of Centro de Investigaciones Biológicas Margarita Salas (Madrid). Human adrenal carcinoma SW13/cl.2 cells were the generous gift of Dr. A. Sarriá (University of Zaragoza, Spain)^41^. SW13/cl.2 cells stably expressing vimentin wt and RFP as separate products (RFP//vimentin wt) have been previously described^6,9^. Cells were cultured in DMEM supplemented with 10% (v/v) FBS and antibiotics (100 U/ml penicillin, 100 μg/ml streptomycin). Stably transfected cell lines were maintained in the presence of 500 μg/ml geneticin. Human lung adenocarcinoma A549 cells (ATCC, CCL-185) were cultured in RPMI1640 with 10% (v/v) FBS, 50 U/ml penicillin, 50 μg/ml streptomycin and 50 μg/ml gentamycin. HAP1 cells are near haploid human cells derived from a chronic myelogenous leukemia cell line, which present an adherent epithelioid-like phenotype. HAP1 cells, parental (vimentin positive, vim +), and vimentin-depleted (vim −) through CRISPR-CAS9-engineering, (reference number HZGHC003297c010) were from Horizon. HAP1 cells were cultured in DMEM-F12 with 10% (v/v) FBS, 100 U/ml penicillin, 100 μg/ml streptomycin, and 2 mM glutamine (Gibco).

### Immunofluorescence studies

Cells were grown on glass coverslips. In the basic immunodetection protocol, coverslips were washed with cold PBS and transferred to a plate cover lined with Parafilm placed over an ice bed and kept at 4 °C, where all incubations and washing steps, with ice cold PBS, were performed. Cells were incubated with Spike protein constructs (10 μg/ml final concentration) in 1% (w/v) BSA in PBS for 1 h. Unless specified otherwise, 1:200 (v/v) antibody dilutions in 1% (w/v) BSA in PBS were routinely used in 1 h incubations. Controls of all secondary antibodies, alone and in combination, as well as others omitting particular primary or secondary antibodies, were performed to ensure specificity of the signals detected. When a particular reagent was omitted, cells were incubated with 1% (w/v) BSA in PBS for an equivalent time. Whenever possible, observations were confirmed using different primary antibodies against the same protein or tag. For CTXB labeling, cells on coverslips were incubated with 0.25 μg/ml CTXB-Alexa555 conjugate in 1% (w/v) BSA in PBS for 10 min either at the beginning or at the end of the procedure involving incubation with Spike constructs plus immunodetection. Incubation and washes were also carried out on ice to avoid toxin endocytosis. Finally, cells were fixed with 4% (w/v) paraformaldehyde (PFA) for 25 min on ice. After washing with PBS and water, coverslips were allowed to dry at room temperature and mounted onto glass slides using Fluorsave (Calbiochem). Where indicated, the fixation step preceded vimentin immunodetection.

In addition, several alternative protocols were used for detection of intracellular proteins or markers of primary cilia. In some assays, fixation with 4% or 2% (w/v) PFA for 15 min at room temperature was preferred, followed by incubations with 0.1% (v/v) Triton X-100 for 5 min, 50 mM glycine for 20 min, and blocking with 1% (w/v) BSA in PBS for 1 h before immunodetection by 1 h incubations at 37 °C with anti-acetylated tubulin (1:800 dilution), or anti-vimentin and anti-ARL13B (1:400 dilution), followed by the corresponding secondary antibodies (1:250 dilution). When indicated, cells were incubated with Spike proteins for 90 min at 37 °C in complete medium before immunodetection. Nuclei were stained with 4,6-diamidino-2-phenylindole (DAPI) from Sigma, at 3 μg/ml in PBS. Variations in these procedures are specified in the corresponding figure legends. Additional technical details, controls and limitations of the tools employed are available as preprint^42^.

### Fluorescent labeling of Spike S1-Fc

A 10 μl aliquot of Spike S1 (1 mg/ml) as provided by the manufacturer was incubated for 20 min at room temperature in the presence of 10- or 7-fold molar excess of FITC or Alexa Fluor 488 carboxylic acid succinimidyl ester (CASE) dye (ThermoFisher Scientific), respectively. Afterwards, excess reagent was removed by filtration through a Zeba desalting micro-spin column (ThermoFisher Scientific). The filtrate was stored at − 80 °C until used.

### Confocal microscopy and image analysis

Images were acquired on SP5 or SP8 Leica confocal microscopes, using 63 × or 100 × oil immersion objectives. Single confocal sections were taken every 0.5 μm in sequential mode. Images were analyzed using LasX or ImageJ software. For analysis of colocalization of the signals of interest at the plasma membrane, band-like regions of interest (ROIs) extending 2 µm outwards from the internal side of the plasma membrane were defined for individual cells in single z-sections with the highest membrane-associated signal, typically, at mid-height of the cell. For colocalization of Spike and vimentin at either the basal (scant cilia) or top (cilia-enriched) regions of the cell, sections within the lower or upper thirds of the cell, respectively, were analyzed. In both cases, ROIs comprising the whole cell area or the area corresponding to the position of cilia, determined with an anti-acetylated tubulin antibody, were compared. Colocalization was analyzed using Fiji’s JACoP plugin and was presented as percentages of coincidental signals (Manders’ coefficient), using thresholds determined either manually or by Costes’ method, as indicated. Colocalization masks were obtained with LasX software.

### Cell lysis and western blot

Cell monolayers were washed with ice-cold PBS. For analysis of vimentin, HAP1 and SW13/cl.2 cells were homogenized in 50 mM Tris–HCl pH 7.5, 0.1 mM EDTA, 0.1 mM EGTA, 0.1 mM β-mercaptoethanol, 0.5% (w/v) SDS, 20 mM sodium orthovanadate, 50 mM sodium fluoride, containing protease inhibitors (2 μg/ml each of leupeptin, aprotinin and trypsin inhibitor, and 1.3 mM Pefablock). For assessment of ACE2 levels, A549 and Vero cells were lysed in RIPA containing cOmplete Protease Inhibitor Cocktail (Sigma). Cell debris was removed by centrifugation at 16,000×*g* for 5 min at 4 °C. Protein concentration was determined by the Bicinchoninic acid method (Pierce, ThermoFisher Scientific). Aliquots of lysates containing 30 μg of protein were separated on SDS-PAGE and transferred to Immobilon-P membranes, essentially as described^43^. After blocking with 2% (w/v) non-fat dried milk, blots were incubated with primary antibodies, typically at 1:500 dilution, followed by HRP-conjugated secondary antibodies, at 1:2000 dilution. In all cases, polypeptides of interest were visualized with the enhanced chemiluminiscence system (ECL, GE Healthcare). Total protein on blots was stained with Simply Blue Colloidal Coomassie reagent (Invitrogen).

### Statistical analysis

Statistical analyses were performed with GraphPad Prism 5 software. Results are shown as mean values ± standard error of the mean (SEM). The colocalization extent upon the different immunofluorescence incubation protocols was statistically compared, for every cell type, by ANOVA with Tukey’s post-test. Non-significant (ns, *p* > 0.05) and significant (*, *p* ≤ 0.05; **, *p* ≤ 0.01; ***, *p* ≤ 0.001) differences are indicated in the graphs.

### Ethics approval

The study has been approved by the CSIC Ethics Committee, reference 063/2020.

## Results

### Detection of Spike protein constructs at the cell surface

**Figure 1:**
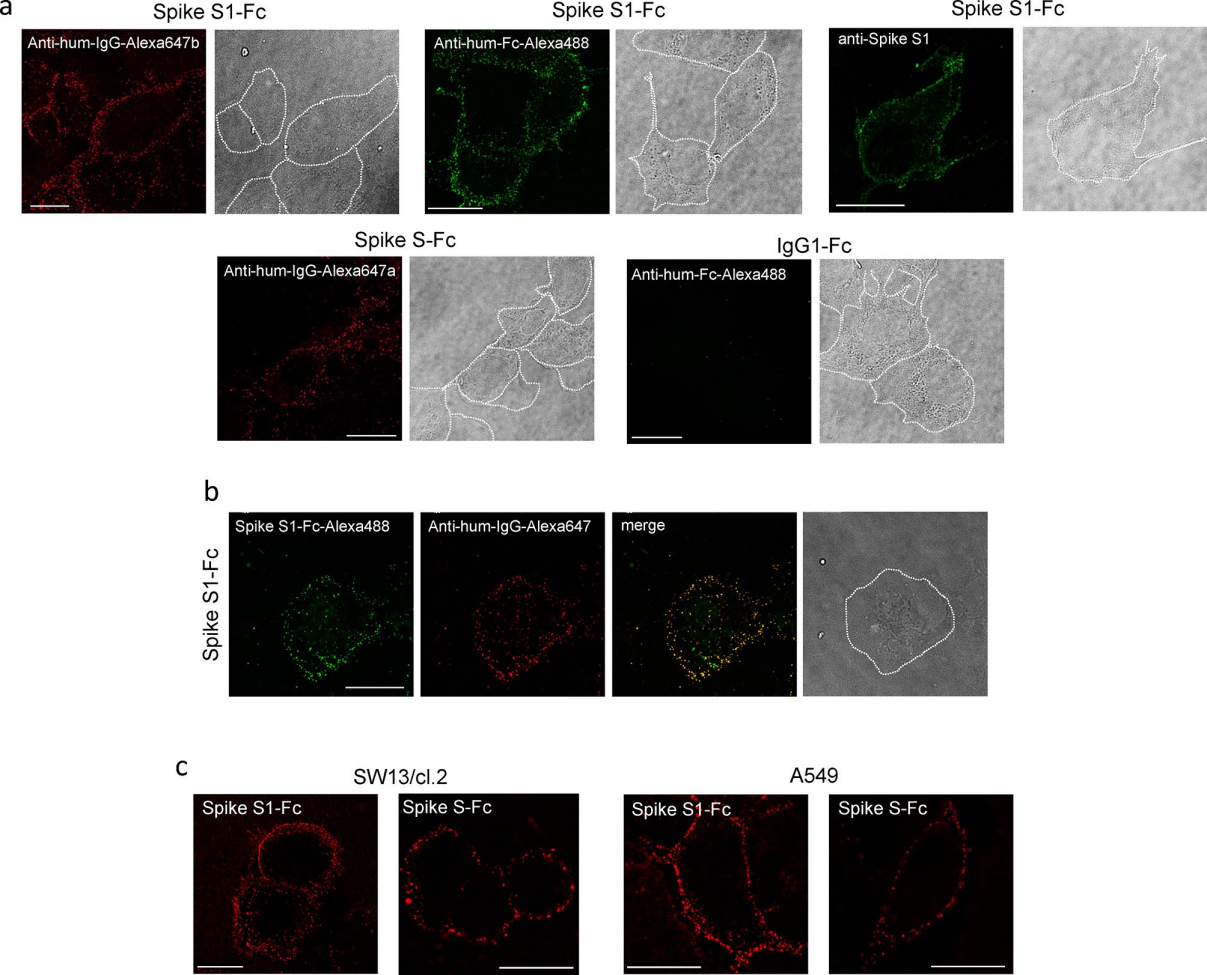
Detection of Spike protein constructs at the cell surface. (**a**) Vero cells plated on glass coverslips were incubated with recombinant Spike S1-Fc, Spike S-Fc, or IgG1-Fc, as indicated, for 1 h in the cold, as described in “Methods”, and the proteins retained on the cell surface were detected with the indicated antibodies. Anti-human (anti-hum) IgG-Alexa647a and Anti-human-IgG-Alexa647b are antibodies raised in goat and alpaca, respectively. (**b**) Cells were incubated with Spike S1-Fc labeled with Alexa488 (green) followed by anti-human IgG-Alexa647 (red) and the overlay of the two signals is shown (merge). In (**a**,**b**), bright field images in which the approximate cell contours have been highlighted are shown. (**c**) SW13/cl.2 or A549 cells were incubated with Spike S1-Fc or Spike S-Fc, which were detected by incubation with anti-human-IgG-Alexa647. Images shown are single confocal sections taken at mid-height of the cells. Bars, 20 μm.

Incubation of Vero cells with Spike S1-Fc protein and subsequent detection by immunofluorescence were carried out on ice to avoid internalization of the viral protein. Spike bound to the cell surface was clearly distinguished as a dotted pattern by confocal microscopy using several Alexa-conjugated anti-human IgG or anti-Fc antibodies (Fig. 1a and Suppl. Figure 1a). Cell-bound tagged Spike S1 was also visualized with an anti-Spike antibody, which gave a clean background in cells incubated with vehicle (Fig. 1a and Suppl. Figure 1b). Additionally, incubation with a Spike S-Fc construct, bearing the S1 and S2 domains, gave a similar cell surface pattern (Fig. 1a). Control incubations performed with the IgG1-Fc fragment resulted in negligible staining, thus excluding a putative role of the Fc tag in the cellular binding of Spike S1-Fc constructs (Fig. 1a). In all cases, controls carried out with the anti-IgG or anti-Fc conjugates gave only background staining (Suppl. Figure 1). Incubation with a fluorescent Spike S1-Fc protein labeled with CASE-Alexa488 followed by direct visualization and immunofluorescence allowed further confirmation of cell binding and the typical dotted pattern at the cell surface (Fig. 1b). Moreover, overlapping of both signals confirmed the specificity of the detection (Fig. 1b).

Binding of Spike proteins to the cell surface was also evidenced in adrenal carcinoma SW13/cl.2 and lung adenocarcinoma A549 cells (Fig. 1c), the latter expressing variable levels of ACE2 protein^44^ but lacking Fc receptors^45^. Importantly, Spike S and S1 protein constructs could still be detected after several hours of incubation on ice and extensive washing, indicating high binding stability. Notably, they were unevenly distributed in the cell population, with more intense staining in some cells or cell areas.

### Spike protein constructs display scarce colocalization with cholera toxin B-positive membrane domains

**Figure 2:**
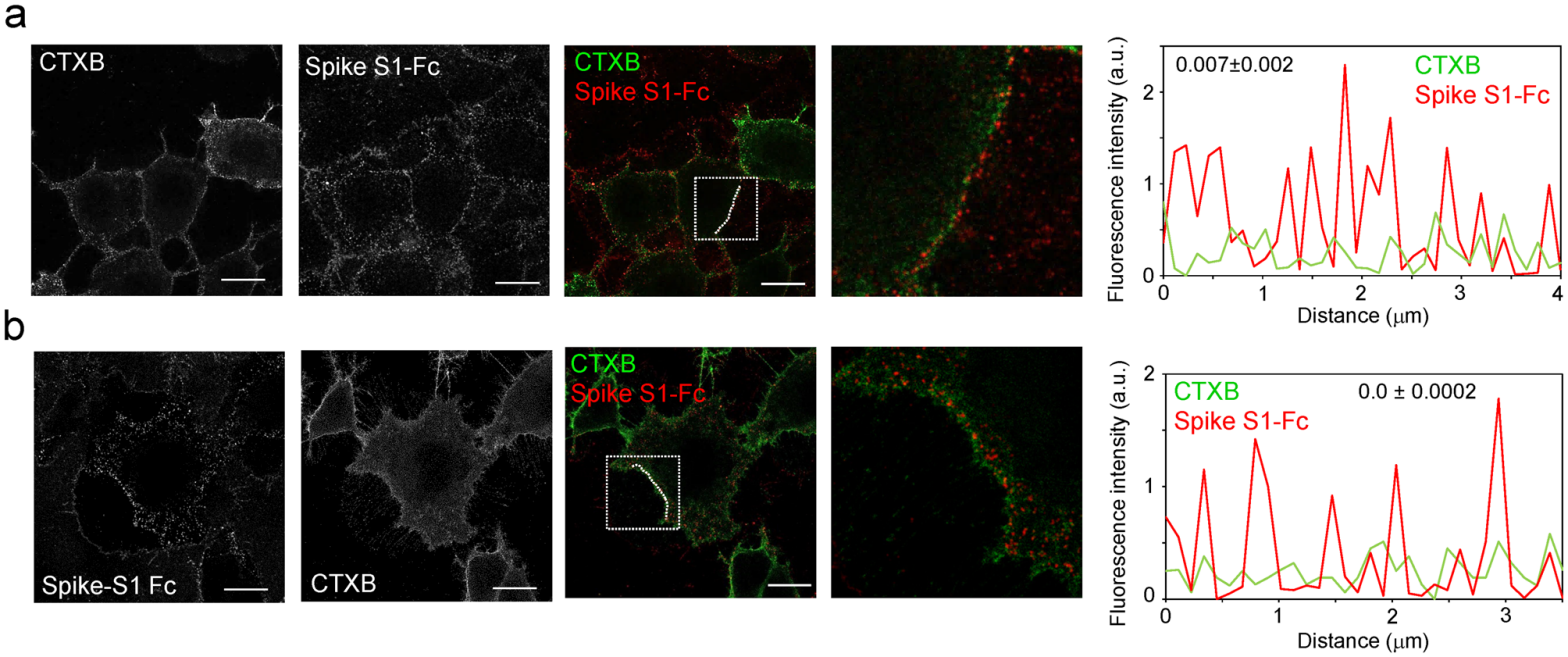
Detection of Spike S1 and cholera toxin-B binding sites in cells. Vero cells on glass coverslips were incubated in the cold with cholera toxin-B–Alexa555 (CTXB) for 10 min before (**a**) or after (**b**) incubation with Spike S1-Fc for 1 h followed by anti-human IgG-Alexa647 for another hour. Cells were extensively washed after each incubation. At the end of the procedure, cells were fixed and mounted for visualization by confocal microscopy. Images of individual channels and a merged image of a section at mid-cell height are shown. Regions of interest in the merged images, delimited by dotted squares, are magnified on the right. Graphs on the far right display the fluorescence intensity profiles of Spike S1-Fc (red) and CTXB (green) signals along the dotted lines marked in the merged images. Numbers in insets show the Manders’ colocalization coefficients for every condition, obtained applying the Costes’ threshold. Results are depicted as mean values ± SEM of 42 and 14 measurements for (**a**,**b**), respectively. Bars, 20 μm.

Several studies have proposed the importance of lipid rafts in SARS-CoV-2 infection. Therefore, cells were preincubated with fluorescent cholera toxin B subunit (CTXB), a widely used marker for membrane microdomains with the characteristics of lipid rafts^46^, which binds the lipid membrane component GM1 ganglioside, and incubated with Spike S1-Fc. CTXB-Alexa555 stained regions or discrete patches of the membrane (Fig. 2a), showing scarce colocalization with the Spike S1-Fc signal, according to Manders’ colocalization coefficient. Nevertheless, the fluorescence intensity profiles showed partial overlap of Spike fluorescent spots and CTXB-positive patches (3 out of 13 Spike S1-Fc-positive peaks coincided with CTXB fluorescence peaks in the representative profile shown in Fig. 2a). CTXB is known to bind up to five molecules of its lipid receptor, thus, associating with and crosslinking lipid rafts, potentially remodeling the underlying membrane^46^. To discard potential effects of CTXB per se, cells were labeled with the toxin after incubation with Spike-Fc protein constructs (Fig. 2b). Again, a virtually negligible colocalization extent was observed, with some Spike S1-Fc-positive peaks coinciding with CTXB peaks in the fluorescence profiles (Fig. 2b). Of note, some apparently intracellular patches of CTXB with scarce colocalization with Spike S1-Fc could also be detected. Therefore, under our conditions, binding of Spike S1 to the cell surface did not show lipid raft specificity.

### Detection of vimentin at the cell surface

Cell surface exposure of vimentin has been reported in several cell types in association with cell senescence and/or oxidative damage^47^ (reviewed in^12^). Nevertheless, the disposition of the protein at the cell surface and the epitopes exposed are not fully elucidated and seem to depend on the experimental model^23,48^. Here we have assessed the presence of vimentin immunoreactive signals at the surface of several cell types, employing a panel of anti-vimentin antibodies, including some “knockout validated” (Supplementary Table 1).

**Figure 3:**
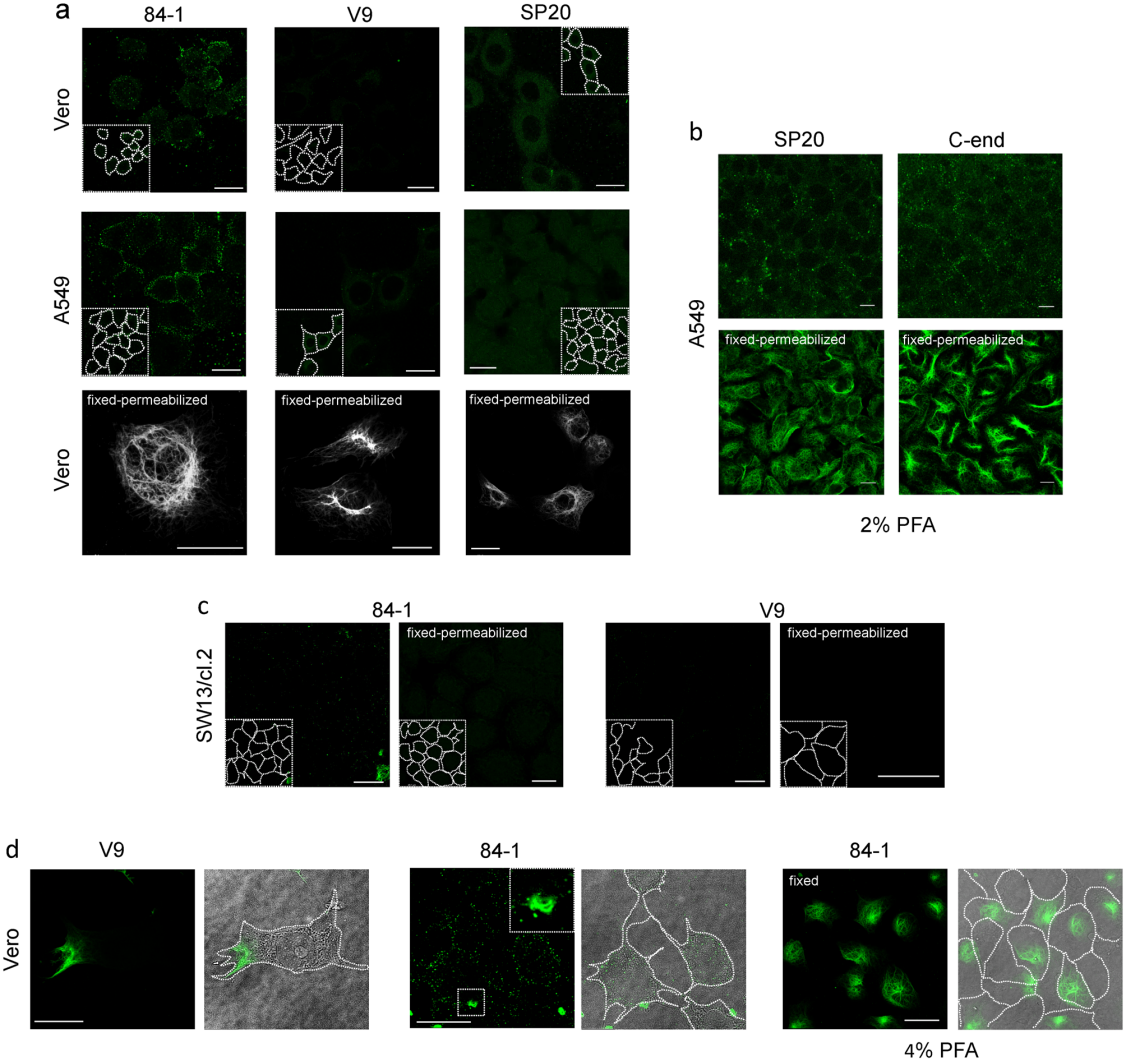
Detection of vimentin in several cell types by several fixation and permeabilization protocols. When indicated, cells were fixed, or fixed and permeabilized, prior to immunodetection. (**a**) In the two upper rows, Vero or A549 cells were incubated with the indicated monoclonal primary antibodies at 1:200 dilution for 1 h in the cold, followed by incubation with Alexa-488-conjugated secondary antibodies at 1:200. At the end of the procedure cells were fixed with 4% (w/v) PFA in the cold. Representative images at mid-cell height are shown. In the lower row, Vero cells were fixed and permeabilized prior to detection of cytoplasmic vimentin with the same monoclonal antibodies. (**b**) A549 cells were incubated with the indicated anti-vimentin antibodies, and the corresponding secondary antibodies, before fixation (upper panels) or after fixation with 2% (w/v) PFA and permeabilization (lower panels) for detection of cytoplasmic vimentin. (**c**) Vimentin-deficient SW13/cl.2 cells, were incubated with the indicated anti-vimentin monoclonal antibodies and Alexa-488-conjugated anti-mouse immunoglobulins at 1:200, before fixation (left images) or after fixation and permeabilization (right images). (**d**) Vero cells were incubated with the indicated anti-vimentin antibodies as described above for detection of cell surface vimentin. Images shown illustrate representative cases of cells in which an area of the cytoplasm shows staining of filamentous vimentin (left panels), or that display fragments of vimentin filaments associated to their surface (middle panels). In the right panels, Vero cells were stained with anti-vimentin antibodies after fixation with 4% (w/v) PFA in the cold without performing any additional permeabilization step. In each case, images on the right show the overlay of the bright field and fluorescence images with the contours of the cells highlighted. Insets in (**a**,**c**) display the approximate cell contours drawn from bright field or overexposed images, whereas the inset in (**d**) middle panels shows a cell surface-associated vimentin bundle. Bars, 20 μm in (**a**,**c**,**d**) and 10 μm in (**b**).

The 84-1 “cell-surface vimentin” antibody^49^, putatively recognizing the beginning of the rod domain^50^, gave a consistent punctate pattern at the cell surface in non-permeabilized Vero and A549 cells (Fig. 3a). This staining was not homogeneous in the cell population, but clearly distinct from the background signal obtained with the secondary antibody (Suppl. Figure 2a). In contrast, very faint signals were obtained with monoclonal antibodies V9 (Fig. 3a) and clone 13.2 (Suppl. Figure 2a), which respectively recognize the vimentin C-terminal “tail” domain and a non-characterized epitope outside the tail domain^9^. Of note, the SP20 antibody, of undefined epitope, yielded diverse patterns at the cell periphery of non-permeabilized Vero or A549 cells depending on the fixative concentration used; a nearly negligible signal with 4% (Fig. 3a), but a peripheral dotted signal with 2% (w/v) PFA (Fig. 3b). Under these conditions, a similar punctate peripheral pattern was also obtained with a goat polyclonal antibody against the C-terminal end of vimentin (C-end, Fig. 3b). All antibodies were able to recognize filamentous vimentin by conventional immunofluorescence protocols employing fixed and permeabilized cells (Fig. 3a,b, lower panels). This ruled out that the faint detection of extracellular vimentin could be due to poor antibody performance.

Specificity of detection was explored with the 84-1 and V9 antibodies in the vimentin-deficient cell line SW13/cl.2 (Fig. 3c). In these cells, 84-1 and to a lesser extent V9, gave a faint irregular dotted background in non-permeabilized cells (Fig. 3c, left panels) and did not detect any filamentous vimentin in fixed and permeabilized cells (Fig. 3c, right panels). These same antibodies were used on HAP1, parental (vim +), and vimentin-depleted (vim -) cells. Parental HAP1 (vim +) cells displayed a robust 84-1 immunoreactive signal consisting in bright dots or small aggregates at the cell periphery, and a faint peripheral signal with V9 (Suppl. Figure 2c). In contrast, HAP1 (vim -) cells showed a low, although non-negligible punctate background with 84-1, and a virtually undetectable V9 signal (Suppl. Figure 2c). Importantly, after permeabilization, both antibodies detected filamentous vimentin only in HAP (vim +) cells. Weak intracellular dotted patterns were observed in scattered cells, which could represent partially damaged cells. Controls including only secondary antibodies gave a negligible or diffuse background in all cell lines (Suppl. Figure 2). Additionally, no bands were detected with the V9^9^ or the 84-1 antibodies in total lysates from vimentin-deficient cells by western blot (Suppl. Figure 2b).

Altogether, among several anti-vimentin antibodies yielding a punctate pattern at the cell surface, the 84-1 antibody showed a more defined distribution, acceptable background and better specificity by various techniques, including dot blot (Suppl. Figure 5), for which it was preferentially used in subsequent experiments.

### Additional phenomena leading to vimentin detection at the cell surface

Notably, in most preparations of Vero or A549 cells, a low proportion of cells showed staining of small areas of cytoplasmic vimentin underneath the plasma membrane (Fig. 3d, left panels). This effect could be due to spontaneous or procedure-induced focal membrane damage, which could allow partial internalization of the antibody. Additionally, small vimentin particles or filament fragments were detected in the extracellular medium or adhered to the surface of some cells (Fig. 3d, middle panels), which could represent released vimentin. Notably, selective detection of cell surface vimentin was found incompatible with prior fixation with 4% (w/v) PFA, which elicited extensive permeabilization in a substantial proportion of cells (Fig. 3d, right panels). Hence, several phenomena related to cell damage may contribute to the presence and detection of vimentin on or at the cell surface by a variety of techniques. Of note, cell culture in the absence of serum did not reduce vimentin staining in A549 cells, or background in vimentin-deficient SW13/cl.2 cells (Suppl. Figure 3), indicating that serum proteins do not constitute an important source of vimentin immunoreactivity under the conditions employed.

### Detection of ACE2

**Figure 4:**
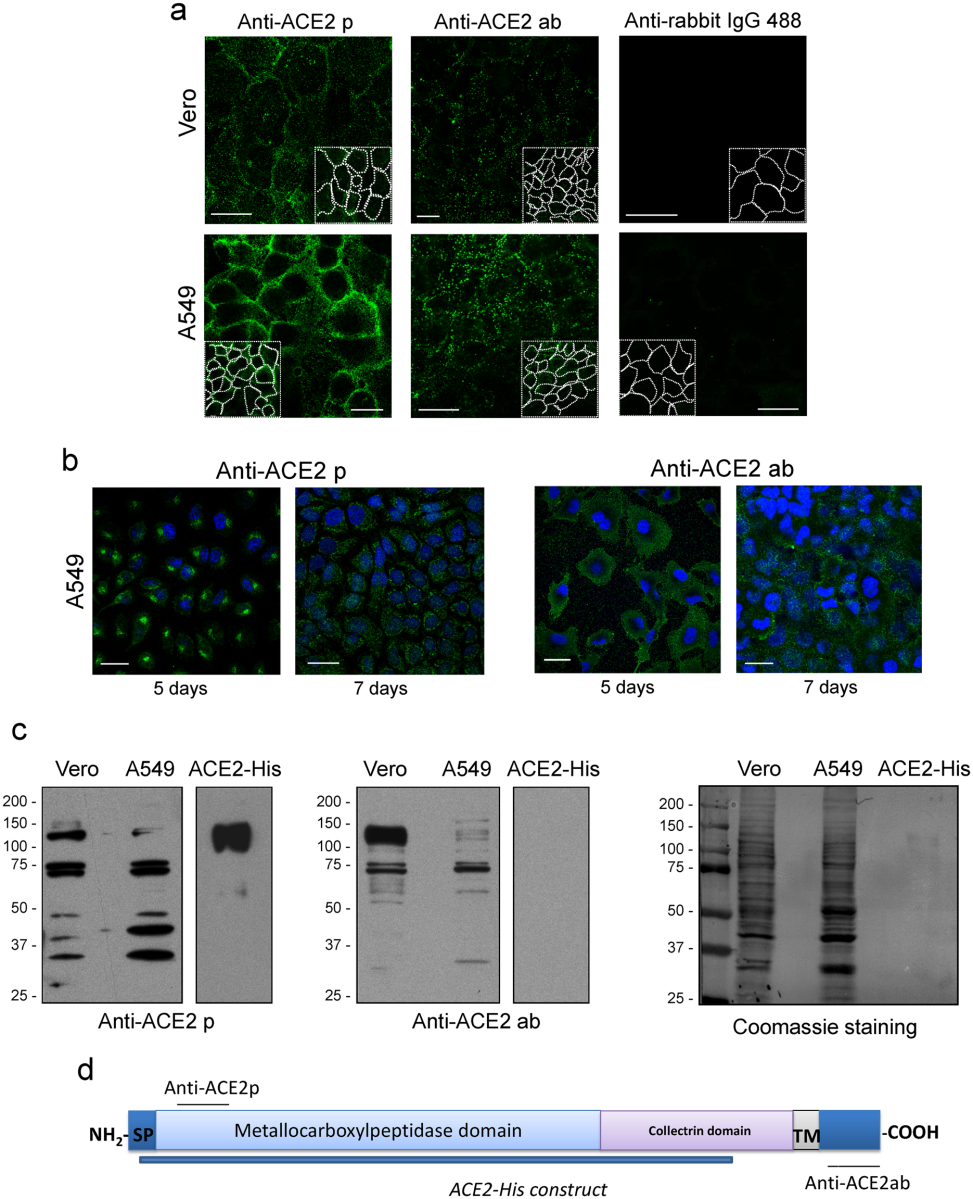
Immunodetection of ACE2. (**a**) Vero or A549 cells were incubated with the indicated anti-ACE2 antibodies prior to fixation. Right panels show the background of the secondary anti-rabbit IgG antibody. Insets show the cell contours. (**b**) A549 cells cultured for 5 days or 7 days after plating were stained with the indicated anti-ACE2 antibodies after fixation with 4% (w/v) PFA and permeabilization with 0.1% (v/v) Triton X-100 for 5 min. Nuclei were counterstained with DAPI. (p, rabbit polyclonal; ab, rabbit polyclonal antibody from Abcam). Bars, 20 μm. (**c**) Detection of ACE2 by western blot. Total cell lysates from the indicated cell types containing 30 μg of protein, or 100 ng of purified ACE2 protein (ACE2-His) were analyzed by SDS-PAGE followed by immunoblot with the indicated anti-ACE2 antibodies. Left panels show a long and a short exposure of the blot of cell lysates and recombinant protein, respectively. Middle panels show a short exposure in both cases. In the right panel, the total protein on blots was visualized by staining with Simply Blue. (**d**) Scheme depicting the sequence of ACE2, the location of the epitopes of the antibodies used and the region spanned by the recombinant protein. SP, signal peptide; TM, transmembrane domain.

ACE2 has been extensively reported to act as a receptor for SARS-CoV-2 and to directly interact with its Spike protein both in vitro and in cells^37,51^. However, as studies on the cellular localization of endogenous ACE2 are scarcer, we attempted its detection in several cell types. A clear staining at the cell periphery of non-permeabilized Vero and A549 cells was obtained with a polyclonal antibody against the N-terminus of the protein (anti-ACE2 p) (Fig. 4a). Unexpectedly, a polyclonal antibody against the carboxyl-terminal region (anti-ACE2 ab), corresponding to the cytoplasmic domain^52^, yielded a yet unexplained peripheral dotted pattern in non-permeabilized cells (Fig. 4a), which was poorly defined but clearly distinguishable from the background of the secondary antibody. In permeabilized A549 cells cultured for 5 days after passage, anti-ACE2 p detected mainly juxtanuclear structures compatible with Golgi localization, but a more disperse cytoplasmic distribution was observed after 7 days of culture (Fig. 4b). In turn, anti-ACE2 ab showed a more diffuse pattern. Both antibodies detected several bands, consistent with ACE2, in western blots of total lysates from Vero and A549 cells, although with different efficiency (Fig. 4c). These bands likely represent different posttranslationally processed forms of the protein, as recently characterized^53^. A broad 120 kDa band in Vero and a doublet in A549 lysates could be detected by anti-ACE2 p but not by anti-ACE2 ab (Fig. 4c), consistent with the C-terminal end specificity of the latter (Fig. 4d). These bands migrated similarly to commercial ACE2 glycosylated protein (Gln^18^-Ser^740^), purified from HEK293 cells (ACE2-His) (Fig. 4c). Both antibodies recognized a doublet of approximately 75 kDa, consistent with the ACE2 peptide spanning residues 130-805, in the two cell types, whereas smaller products, likely representing cleaved forms, were preferentially recognized by anti-ACE2 p. These recognition patterns are consistent with the diverse cellular ACE2 forms reported and the localization of the respective antibody epitopes (Fig. 4d). Taken together, these results indicate that anti-ACE2 p and anti-ACE2 ab antibodies are able to detect ACE2 through various techniques, although the different patterns observed may depend on their ability to recognize distinctly processed ACE2 forms.

### Detection of Spike protein constructs and vimentin on the surface of cells

**Figure 5:**
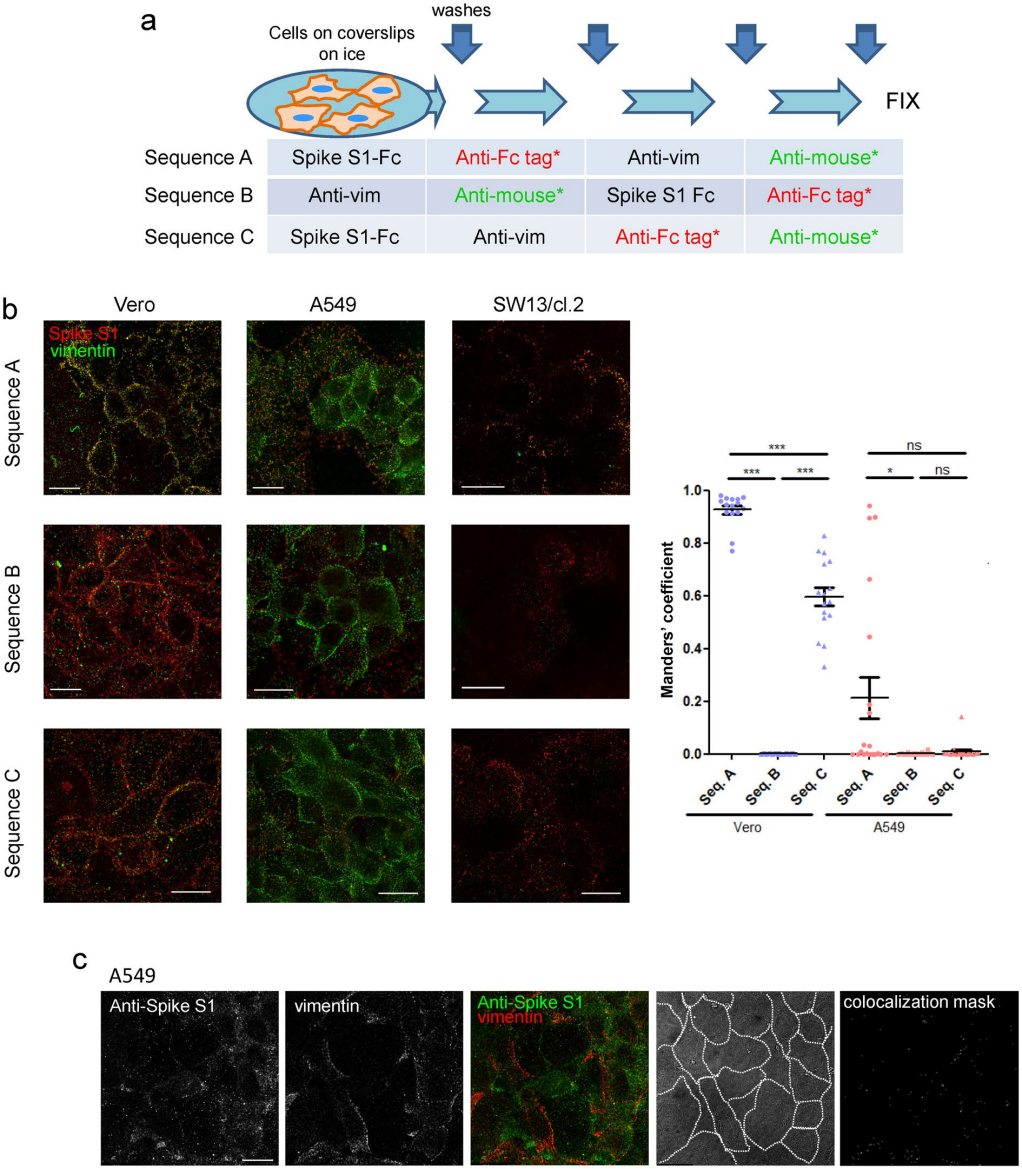
Detection of Spike S1 and vimentin in several cell types employing different immunodetection sequences. (**a**) Scheme of the incubation and washing steps performed for immunodetection. Incubations were carried out for 1 h in the cold. After each incubation coverslips were washed three times with 200 μl of cold PBS. At the end of the procedure, an additional washing step with water was performed before coverslips were allowed to dry and mounted. (**b**) Representative images from the detection of Spike S1-Fc and vimentin in the indicated cell lines following the different immunodetection sequences. The graph shows the colocalization between Spike and vimentin fluorescent signals for Vero and A549 cells with every immunodetection sequence assayed. Colocalization is expressed as the proportion of Spike S1 colocalizing with vimentin signal (Manders’ coefficient), measured applying the automatic Costes’ threshold (n ≥ 12 per condition). Results are shown as mean values ± SEM; ns, non-significant, *p* > 0.05; *, *p* ≤ 0.05; **, *p* ≤ 0.01; ***, *p* ≤ 0.001 by ANOVA with Tukey’s post-test. (**c**) A549 cells were incubated with Spike S1-Fc, after which, immunodetection was performed by simultaneous incubation with anti-S1 antibody and 84-1 anti-vimentin monoclonal antibody, followed by simultaneous incubation with the two corresponding secondary antibodies prior to fixation. The bright field image with cell contours highlighted and colocalization mask are shown at the right. Bars, 20 μm.

To assess the relative position of Spike positive spots and vimentin immunoreactive signals at the surface of live cells, several sequential immunofluorescence protocols were employed, schematized in Fig. 5a. Briefly, in sequence A, cells were incubated first with Spike S1-Fc and the corresponding anti-human IgG antibody prior to vimentin immunodetection, whereas in sequence B incubations followed the opposite order. In sequence C, cells were incubated first with the primary reagents, i.e., Spike S1-Fc and anti-vimentin, and later with the secondary antibodies. Illustrative examples of images taken at mid-height of the cells are shown in Fig. 5b. Spike/vimentin colocalization was quantified using Manders’ coefficients as the percentage of Spike overlapping with the vimentin immunoreactive signal (Fig. 5b graph). Significant colocalization of Spike S1 and vimentin was found at the surface of Vero cells using incubation sequence A (Fig. 5b, left column). Nevertheless, vimentin staining was weaker and colocalization with Spike was virtually abolished when sequence B was employed , while sequence C rendered an intermediate staining pattern and colocalization extent. Of note, vimentin fragments adhered on the cell surface did not show any consistent enrichment of Spike binding (Suppl. Figure 4).

A549 cells exhibited a similar cell surface vimentin pattern independently of the strategy used for immunodetection (Fig. 5b, middle column images). Nevertheless, the immunodetection sequence also influenced the colocalization extent with Spike S1. In samples processed in sequence A, a partial and widely variable coincidence of Spike and vimentin-positive spots was observed. Curiously, patches of the membrane with more intense or continuous vimentin frequently showed scarce Spike signals. Notably, colocalization at mid-height was virtually blunted when applying sequences B or C. Additionally, vimentin-negative SW13/cl.2 cells, used as an additional control, showed detectable Spike binding (as shown in Fig. 1c) in spite of residual vimentin background staining (illustrated also in Fig. 3c).

Therefore, it appears that the Spike/vimentin colocalization extent is markedly dependent on the cell type and the incubation sequence in the detection protocol.

Several factors could contribute to the higher vimentin signal and/or colocalization with Spike S1 observed in the various cell types using sequence A. Potential crossreactivity between the different reagents employed for detection was addressed by dot blots, in which antibodies gave variable signals with other immunoglobulins (Suppl. Figure 5). Anti-vimentin 84-1 was the primary antibody showing superior specificity in terms of higher vimentin signal and lower crossreactivity, whereas V9 gave a slightly higher background with some immunoglobulins and clone 13.2 yielded a low vimentin signal. Taken together, these observations indicate that, despite the high specificity of some of the antibodies used (i.e., knockout validated), binding to other immune complexes or new epitopes formed during the experiment could occur, for which confirmation of the results with several reagents is advisable.

Hence, the potential colocalization of Spike S1 and vimentin at the surface of A549 cells was evaluated by an alternative protocol using an anti-Spike antibody. The results, shown in Fig. 5c indicate a low extent of Spike S1/vimentin colocalization, as suggested by image analysis (Pearson’s correlation 0.11; overlap coefficient, 0.35; colocalization rate, 12%), and illustrated by the colocalization mask (Fig. 5c).

### Detection of Spike protein constructs and ACE2 on the surface of cells

**Figure 6:**
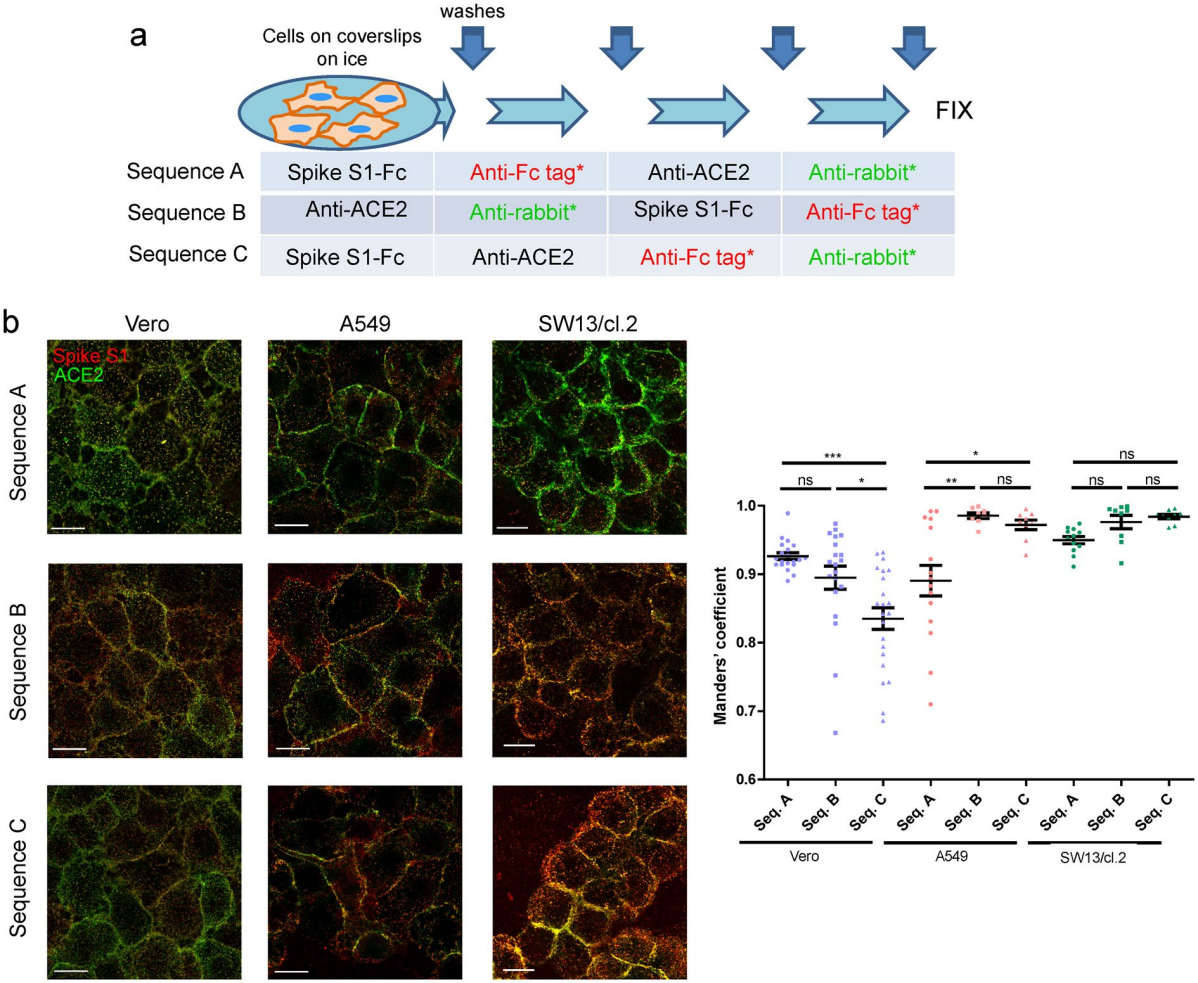
Detection of Spike constructs and ACE2 in several cell types employing different sequences for immunodetection. (**a**) Scheme of the incubation and washing steps performed for immunodetection. Incubations were carried out for 1 h in the cold. After each incubation coverslips were washed three times with 200 μl of cold PBS. At the end of the procedure, an additional washing step with water was performed before coverslips were allowed to dry and and mounted. (**b**) Representative images from the detection of Spike constructs and ACE2 in the indicated cell lines employing the different immunodetection sequences. The graph shows the colocalization between Spike S1-Fc and ACE2 fluorescent signals for every cell type and immunodetection sequence assayed. Colocalization is expressed as the proportion of Spike S1 colocalizing with ACE2 signal (Manders’ coefficient), measured applying the automatic Costes’ threshold (n ≥ 8 per condition). Results are shown as mean values ± SEM; ns, non-significant, *p* > 0.05; *, *p* ≤ 0.05; **, *p* ≤ 0.01; ***, *p* ≤ 0.001 by ANOVA with Tukey’s post-test. Bars, 20 μm.

Similar strategies to those depicted in Fig. 5a, were used to explore the Spike/ACE2 colocalization (Fig. 6a). Anti-ACE2 p gave a clear signal at the periphery of non-permeabilized cells in all cell types tested, regardless of the immunodetection sequence employed (Fig. 6b). Moreover, a high colocalization rate of Spike with ACE2 signals was obtained at mid-cell height in all conditions and cell types (Fig. 6b, graph), and was particularly evident at some cell–cell contacts.

### Detection of ACE2 and vimentin on the surface of cells

**Figure 7:**
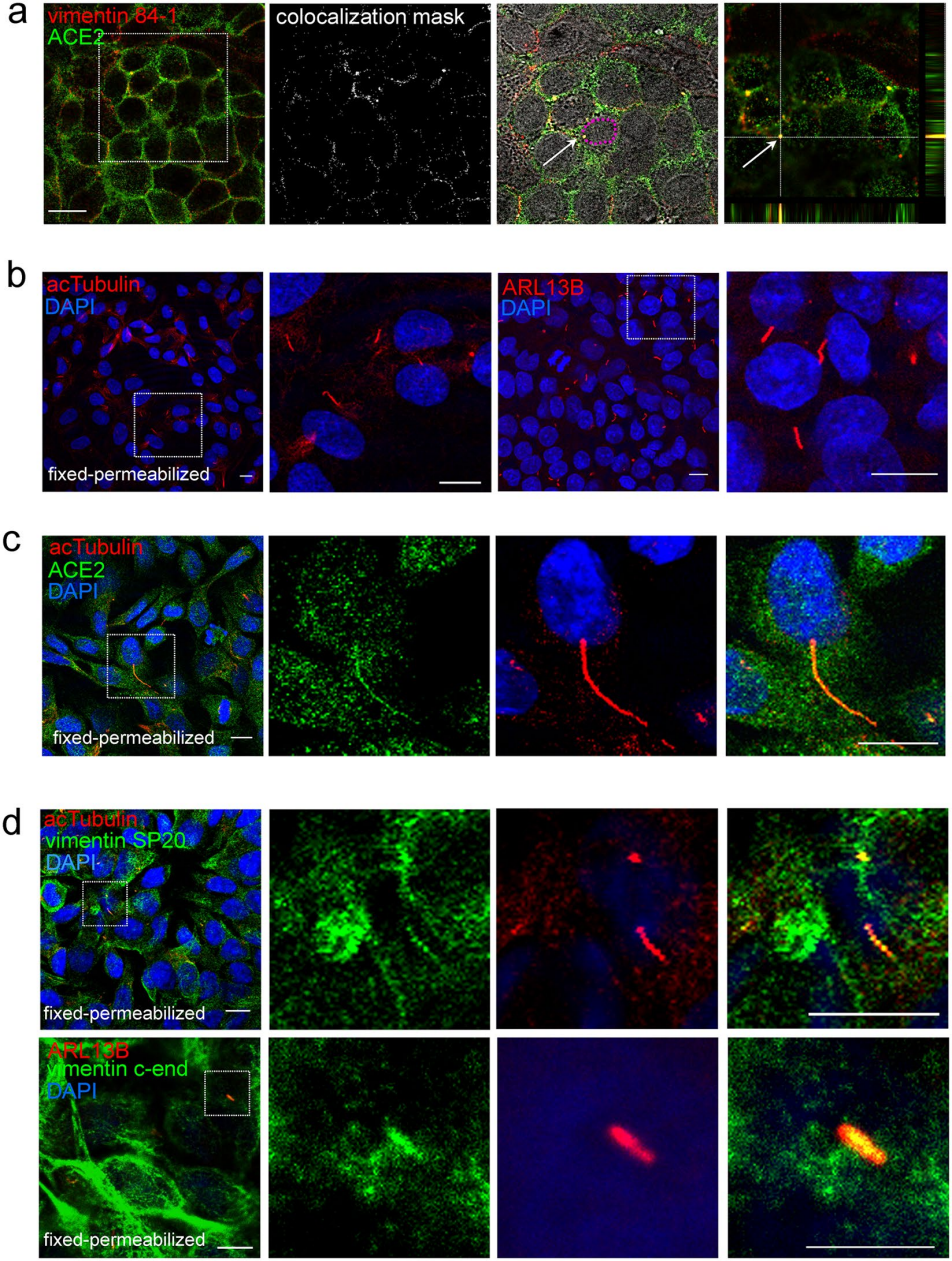
Detection of vimentin and ACE2 in A549 cells. (**a**) Live A549 cells were incubated simultaneously with anti-vimentin and anti-ACE2 antibodies, after which they were incubated with a combination of the corresponding secondary antibodies prior to fixation. An illustrative image is depicted showing points of colocalization at intercellular contacts at mid-cell height, highlighted in the colocalization mask. In addition, an overlay of the region of interest delimited by the dotted square with the bright field image is shown to illustrate the juxtanuclear position of one of the colocalization points (white arrow); the contour of the nucleus is highlighted in magenta. The far right panel shows the top section of the region of interest along with the orthogonal projections centered in the colocalization point marked with the arrow. Bars, 20 μm. (**b**–**d**) Cells were fixed and permeabilized as specified below before immunodetection. (**b**) A549 cells were fixed with 4% (w/v) PFA and permeabilized with 0.1% (v/v) Triton for 5 min for staining with monoclonal antibodies against acetylated tubulin (acTubulin, left panels) or ARL13B (right panels). Nuclei were stained with DAPI. (**c**) A549 cells fixed and permeabilized as in (**b**), were stained with anti-ACE2 and anti-acetylated tubulin antibodies. (**d**) A549 cells were fixed with 2% (w/v) PFA, permeabilized with 0.1% (v/v) Triton X-100 and stained with anti-vimentin antibodies (SP20 or C-end) and either anti-acetylated tubulin or anti-ARL13B antibodies, as indicated. Images in (**b**–**d**) are single sections. In each case, the regions of interest (dotted square) are enlarged at the right. Bars, 10 μm.

In an earlier report, vimentin was identified among the proteins coimmunoprecipitating with ACE2 in Vero E6 cells exposed to SARS-CoV^23^. Here, given the importance of SARS-CoV-2 in lung pathology, we explored the presence of both proteins on the surface of live human cells of lung origin, namely, A549 cells, by immunofluorescence (Fig. 7a). For these assays, cells were incubated with a combination of the two primary antibodies followed by the secondary antibodies. A moderate degree of vimentin/ACE2 colocalization was observed (Pearson’s correlation 0.36; overlap coefficient, 0.64; colocalization rate, 20%). Interestingly, colocalization was especially consistent at some intercellular segments as well as at certain spots, as highlighted in the colocalization mask (Fig. 7a). Remarkably, some of the colocalizing spots appeared close to the nucleus and spanned multiple sections of the cell, as observed in orthogonal projections, being still noticeable at the upper cell layers (Fig. 7a), which indicates that they correspond to elongated structures. Interestingly, ACE2 has been recently shown to localize at the motile cilia of airway epithelial cells^54^, which have been directly implicated in SARS virus infection^54,55^, as well as at the primary cilium of a kidney cell line^54^. Intriguingly, overlap between the signals of vimentin, used as a fibroblast marker, and acetylated tubulin, a known marker of cilia^56,57^, yet unexplored, can be spotted in images from previous studies^58^. This prompted us to monitor the presence of ACE2 and vimentin at primary cilia in A549 cells using several cell culture conditions, sample preparation strategies and cilia markers.

**Figure 8:**
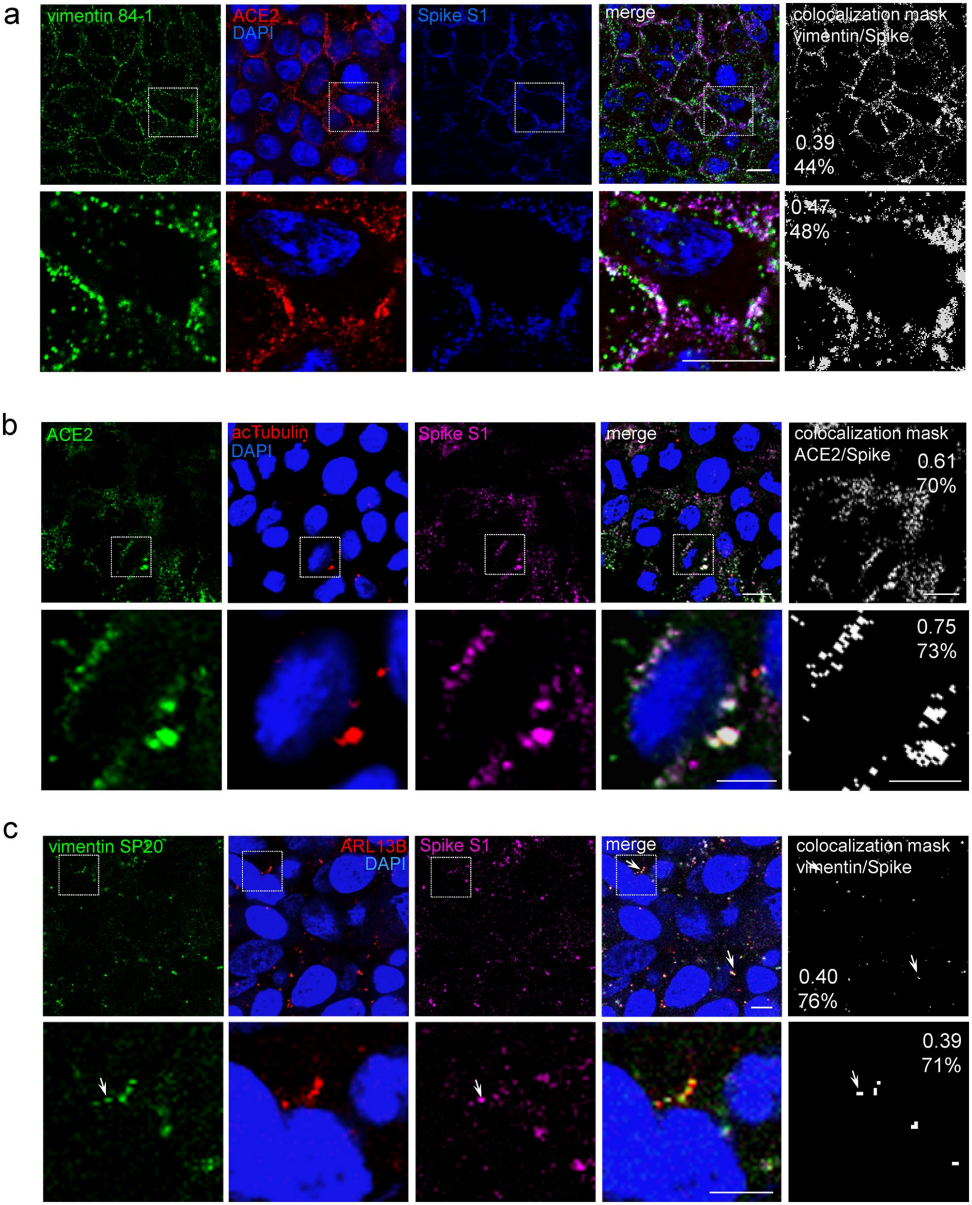
Detection of Spike S1, ACE2 and vimentin at primary cilia. A549 cells were incubated with Spike S1-Fc at 37 °C for 90 min in complete medium. Non-permeabilized cells were stained with (**a**) anti-ACE2 and anti-vimentin 84-1 antibodies, (**b**) anti-ACE2 and anti-acetylated tubulin or (**c**) anti-vimentin SP20 and anti-ARL13B antibodies, followed by the corresponding secondary antibodies, and finally with anti-human IgG antibody to detect the Spike S1 Fc tag, before fixation. In (**a**,**b**) cells were fixed with 4% (w/v) PFA and in (**c**) with 2% (w/v) PFA. Images shown are single sections for each channel obtained by confocal microscopy, at middle height of the cells, and the corresponding overlays. Regions of interest (dotted squares) are enlarged in the lower panels for each condition. Colocalization masks for the signals of (**a**) vimentin and Spike, (**b**) ACE2 and Spike, and (**c**) vimentin and Spike, are shown at the right as white signals on black background; numbers in insets correspond to the Pearson’s coefficient and the percentage of colocalization for the regions shown. Colocalization analysis was performed with Leica software. Arrows in c point to structures showing colocalization. Bars, 10 μm.

Primary cilia could be distinguished in the A549 cell population based on their positive signals for the cilia markers acetylated tubulin and ADP-ribosylation factor-like protein 13B (ARL13B) (Fig. 7b), their elongated appearance and presence as a single structure per cell, frequently stemming from a juxtanuclear position and being detectable at the upper cell layers (Fig. 7b). Interestingly, acetylated tubulin-positive cilia showed frequent ACE2 positive staining (Fig. 7c). Importantly, vimentin immunoreactivity at ACE2 and acetylated tubulin or ARL13B-positive structures with the characteristics of primary cilia could be confirmed with several anti-vimentin antibodies including 84-1, SP20 and C-end, both in permeabilized (Fig. 7d) and non-permeabilized cells (Figs. 7a, 8a,c). Vimentin-deficient SW13/cl.2 adrenocortical carcinoma cells, used a negative control, displayed short structures positive for ACE2 and acetylated tubulin or ARL13B (Suppl. Figure 6), consistent with the imperfect cilia formation of this cell type^59^. Furthermore, these structures lacked vimentin staining, confirming the specificity of vimentin detection at primary cilia shown in Fig. 7d.

### Detection of Spike S1, ACE2 and vimentin at the primary cilia of A549 cells

Finally, we assessed whether the Spike S1 protein bound to primary cilia, using a modification of sequence C (Fig. 8). Live A549 cells were incubated with Spike S1-Fc prior to immunodetection with a combination of anti-ACE2 and anti-vimentin antibodies, followed by the corresponding secondary antibodies and, finally, with anti-human IgG for Spike-S1-Fc detection, before fixation. Under these conditions, vimentin colocalization with ACE2 showed significant overlap (Pearson’s coefficient ~ 0.4; Manders’ coefficient with Costes’ threshold, 0.9). Moreover, certain Spike S1 spots were observed at the cell periphery in close proximity to ACE2 and vimentin signals (Fig. 8a). However, spotting primary cilia on the sole basis of ACE2 immunoreactivity was not straight-forward. Therefore, complementary assays were implemented for staining of Spike S1/ACE2/acetylated tubulin (Fig. 8b) and Spike S1/vimentin/ARL13B (Fig. 8c). Both strategies allowed detection of Spike S1 on cilia in the proximity of ACE2 or vimentin-positive signals, and the corresponding cilia markers. Moreover, image analysis evidenced ACE2/Spike colocalization (Pearson’s coefficient 0.6), and moderate, but clear vimentin/Spike S1 coincidence (Pearson’s coefficient ~ 0.4), as illustrated by the colocalization masks (Fig. 8, right panels). Together, these observations evidence a particular coincidence of Spike S1, ACE2 and vimentin signals at primary cilia.

**Figure 9:**
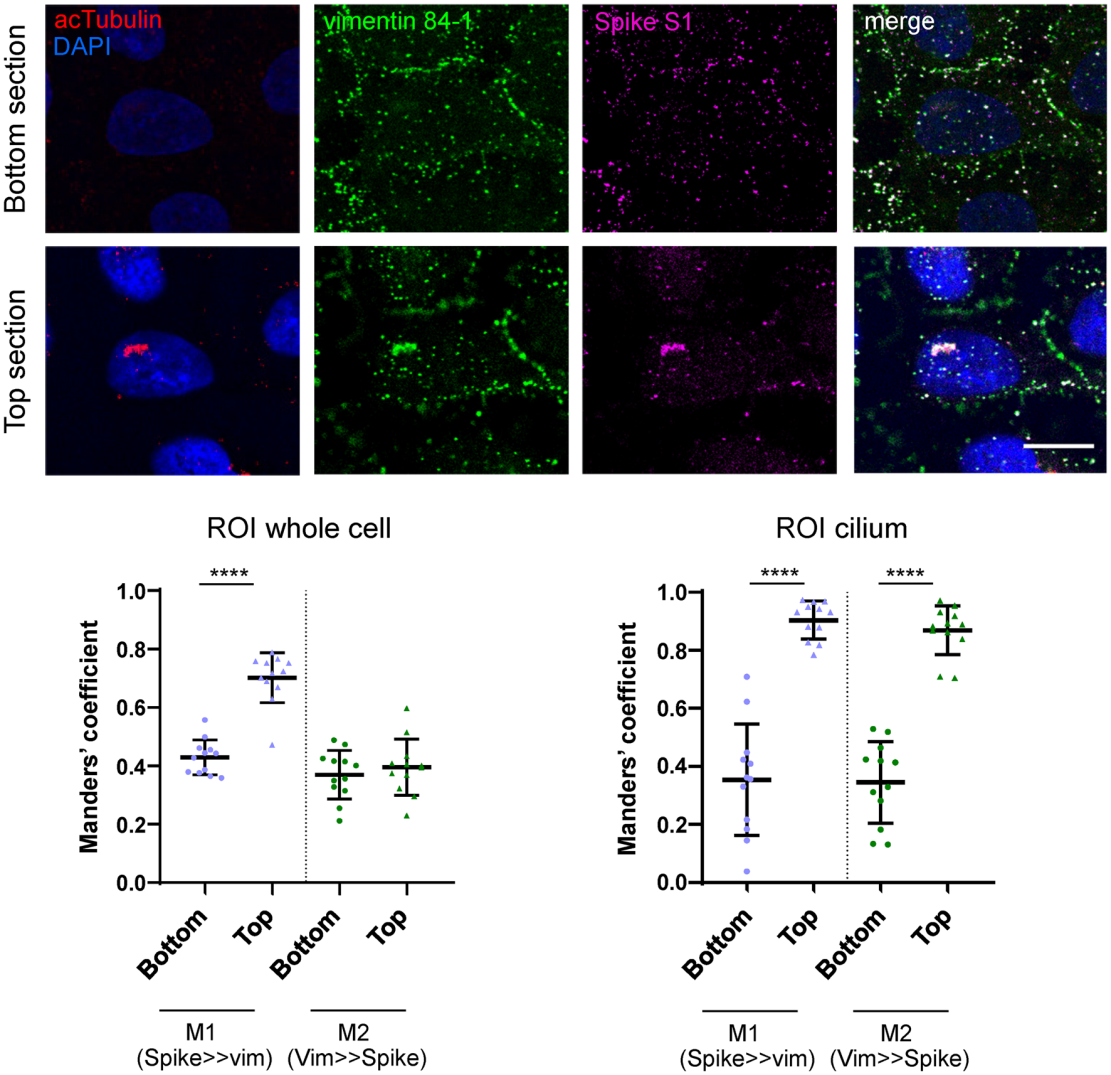
Selective Spike/vimentin colocalization in primary cilia. A549 were incubated with Spike S1-Fc, immunostained as in Fig. 8, and monitored by confocal microscopy. Images shown are single sections taken from the lower (bottom section, absence of cilium) or the upper third (top section, presence of cilium) of the cells, as indicated. Graphs show the Manders’ coefficients for Spike overlapping vimentin (Spike >> vim, M1) and vimentin overlapping Spike (Vim >> Spike, M2) at the basal (circles) and top (triangles) cell sections, obtained considering an individual cell (left graph) or the cilium region (right graph), as ROI. Results are average values ± SEM of 12 determinations from three different experiments. ****, *p* ≤ 0.0001 by unpaired Student’s *t*-test. Bars, 20 μm.

Selectivity of Spike/vimentin colocalization at primary cilia was further substantiated by analysis of basal (scant cilia) and top (cilia-enriched) cell regions (Fig. 9). A significantly higher proportion of Spike overlapping vimentin was measured at the top cell sections, corresponding with the presence of cilia. In contrast, the proportion of vimentin overlaid by Spike was moderate and did not vary significantly between cell sections. When the analysis was restricted to the area occupied by the primary cilia, defined by the acetylated tubulin signal, markedly higher Spike/vimentin colocalization extents were obtained for the top cell layers, as well as an increased proportion of vimentin overlaid by Spike at this structure. Taken together, these results indicate a selective concentration of Spike and vimentin coincident with primary cilia.

## Discussion

The docking of viruses onto cells is a multifaceted process in which multiple viral structures interact with various cellular components with diverse affinities to promote or facilitate viral entry. These interactions often involve cellular elements that cannot be considered true viral receptors since they cannot initiate cytoplasmic viral RNA uncoating, necessary for virus replication^60^. Among the numerous cell membrane-associated structures identified that can contribute to SARS-CoV-2 invasion, ACE2 is the most thoroughly studied. However, involvement of other proteins as viral receptors or co-receptors, including the intermediate filament protein vimentin, has been hypothesized^12,61,62^. The immunodetection studies herein reported indicate a limited overall vimentin/Spike colocalization in non-permeabilized cells, but a consistent vimentin/ACE2/Spike localization at certain structures, especially at the primary cilium, which also appears to be a site for the attachment of SARS-CoV-2 Spike proteins. These observations open new possibilities to study a potential role of vimentin in the modulation of primary cilia and its connections with ACE2 function and viral infection.

Our study employs widely available tools, such as recombinant Spike protein constructs and several commonly used cell lines, to make an initial assessment of vimentin at the cell surface in relation to Spike binding sites. In our models, tagged Spike proteins lined the surface of several cell types yielding a rather homogeneous dotted pattern. Membrane lipid rafts have been proposed as important points for viral entry and potential therapeutic targets^63^. Membrane sites compatible with lipid rafts concentrate ACE2 and other viral entry factors upon cholesterol supplementation^38^; therefore, protective strategies against coronavirus infections through lowering or disrupting membrane cholesterol have been put forward^63,64^. In addition, molecular dynamics simulations have proposed a ganglioside binding surface at the N-terminal domain of the SARS-CoV-2 Spike protein^65^. Nevertheless, the presence of ACE2 in lipid rafts is still controversial^66^ and evidence on the association of SARS-CoV-2 with lipid rafts is mainly indirect. Under our conditions, the low degree of colocalization of Spike constructs with the lipid raft marker CTXB does not support a preferential localization of Spike proteins at these membrane domains. Nevertheless, these results do not exclude the possibility that the virus may interact with lipid rafts in vivo.

Detection of cell surface vimentin is not straight forward since the proteoforms present and the accessibility of its epitopes are still not thoroughly characterized (reviewed in^12,67^). Cell surface vimentin seems to exist in particular non-filamentous assemblies and/or bear certain posttranslational modifications^14,68,69^. Phosphorylation, lipoxidation and/or oxidative modifications have been associated with the presence or increased cell surface exposure or secretion of vimentin in diverse experimental systems^14,47,69^. In addition, fixation procedures, as shown herein, can permeabilize or damage the cell membrane allowing antibody entry and cytoplasmic vimentin staining. This, in turn, can be the source of false positive signals above all in approaches that do not provide imaging, such as ELISA or flow cytometry. Therefore, detection and characterization of cell surface vimentin is a challenging task that requires working with non-permeabilized, ideally non-fixed cells. Published images of cell surface vimentin do not respond to a universal pattern, and diverse arrangements including accumulations^70,71^, cell-membrane associated patches of variable extension^49,72,73^, dotted patterns^49^ and isolated dots^74^ have been reported.

Importantly, detection of cell surface vimentin relies on the availability of specific antibodies that perform adequately in non-permeabilized cells, and do not show crossreactivity with other cellular antigens or reagents in the protocol. Many of the antibodies employed herein have been validated by various methods (Suppl. Table 1), including their lack of signal in vimentin-deficient or knockout cells. Nevertheless, most of them have not been validated for the specific detection of cell surface vimentin. Under our conditions, incubation of live, non-permeabilized cells with a variety of anti-vimentin antibodies gave a dotted pattern unevenly distributed along the cell surface. In addition, the presence of adhered vimentin or of focal membrane permeabilization contributed to the heterogeneity of cell surface vimentin patterns. Although the 84-1 anti-vimentin antibody showed great specificity by western blot and immunofluorescence, a faint background was detected in vimentin-deficient cell lines SW13/cl.2 and HAP1 (vim -). Putative sources of background could be vimentin present in serum, e.g., in exosomes^75^ or minor crossreactivity of antibodies with other structures. Indeed, certain anti-vimentin antibodies generated in patients show crossreactivity with antigens from pathogens, such as streptococcal Hsp70^76^.

Remarkably, the overall Spike/vimentin colocalization varied with the immunodetection sequence, being higher when the Spike construct was detected first (sequence A in Fig. 5). This phenomenon could be due to technical or mechanistic reasons. A high colocalization signal could be due to crossreactivity with the tagged Spike proteins employed, neoepitopes or immune complexes formed during immunodetection. Conversely, prior incubation with Spike and anti-human immunoglobulins could affect cells eliciting focal cell damage and/or vimentin exposure, although this may seem unlikely at 4 °C. Otherwise, prior incubation with anti-vimentin antibodies could shield some Spike docking sites leading to alternative binding. In this context, a recent preprint reported the in vitro interaction between Spike-containing viral-like particles and vimentin, together with a blocking effect of some anti-vimentin antibodies on their uptake in an ACE2-overexpressing cell line^62^. Nevertheless, under our conditions, neither the lateral areas of cells with more intense vimentin signal nor the vimentin fragments adhered to the cell surface showed any particular enrichment of Spike binding.

Consistent with previous reports^77^, immunofluorescence of intact cells with anti-ACE-2 antibodies resulted in a robust punctate staining at the cell periphery in all cell types, including A549 cells. Interestingly, ACE2 levels and/or localization can vary in A549 cells depending on the proliferative state^44^ but, as shown here, the pattern may also change with the degree of cell confluence. Nevertheless, we observed a marked Spike/ACE2 colocalization under the various incubation conditions employed.

Remarkably, ACE2 and vimentin signals displayed an apparently specific colocalization pattern, coinciding at certain intercellular patches and distinct cellular structures compatible with primary cilia. The primary cilium projects from the surface of most mammalian cells and plays key roles in the interaction with the environment and cell cycle control^78^. This structure possesses a core of nine microtubules and a highly complex protein composition^79^. In turn, motile cilia are present at the apical surface of ciliated cells in the airways and other ducts. Notably, motile ciliated cells in the airways have been reported to originate from primary ciliated cells^80^. Interestingly, the presence of ACE2 both at the motile cilia of epithelial airway cells and at the primary cilia of a ciliated kidney epithelial cell line was recently found^54^. However, to the best of our knowledge, the participation of vimentin in cilium architecture or function had not been underscored. Nevertheless, some previous connections between intermediate filaments and primary cilium have been reported, including a role of the intermediate filament-associated proteins trichoplein and filamin A in ciliogenesis^81,82^, as well as the presence of a net of intermediate filaments around basal bodies^83^. Curiously, under our conditions, both ACE2- and vimentin-positive structures in the primary cilium were accessible to immunodetection in non-permeabilized cells in the cold, while fixation and permeabilization allowed their recognition in longer and more numerous cilia. These observations may suggest a partial exposure of these proteins on the cilium membrane or a particular permeability of the membrane at this location.

The presence of ACE2 at cilia has been related to the involvement of these structures as primary points of SARS-CoV-2 docking for entry into cells. Indeed, ciliated cells have been identified as selective targets for SARS-CoV-2 infection in a study on human tissue^54^. However, whereas association of viral particles and ciliated structures has been observed for other coronaviruses^55,84^, a study employing human airway epithelium, found extracellular virions frequently associated with microvilli but rarely with ciliary membranes^85^. Therefore, the role of cilia in SARS-CoV-2 docking is still controversial. Here, we have observed that Spike binding to the top layer of cells concentrates at vimentin-positive regions, coincident with primary cilia, whereas binding to lateral sites displays a moderate colocalization with vimentin. In contrast, Spike/ACE2 colocalization is high at lateral sites as well. Thus, Spike/vimentin colocalization displays improved selectivity at primary cilia. On the other hand, SARS-CoV-2 infection has been proposed to associate with deciliation of invaded cells and cilia and flagellar dysfunction (reviewed in^86,87^). In addition, dedifferentiation of multiciliated cells and loss of motile cilia have been reported after SARS-CoV-2 infection in a reconstructed human bronchial epithelium model, as well as in Syrian hamsters in vivo^88^. Indeed, a recent hypothesis envisages that many of the pathological alterations in COVID-19 could be the result of cilia dysfunction^86^. In this context, although neither the function of ACE2 or vimentin in cilia is known, it would be interesting to study their potential involvement in SARS-CoV-2-induced cilia alterations. In the light of recent findings proposing vimentin as an attachment factor for SARS-CoV-2^89,90^, a positive role of this protein in the docking of the virus onto cilia, therefore contributing to cilia dysfunction, could be speculated. Nevertheless, whether exogenous extracellular vimentin can play a beneficial or deleterious role in viral invasion remains a matter of controversy^89,90^.

## Concluding remarks

Vimentin has been reported to influence cell susceptibility to bacterial and viral pathogens, either in a positive or in a negative way, through various mechanisms, including behaving as an attachment factor at the cell membrane or influencing the replicative cycle^12,20,25,91,92^. The potential role of vimentin in SARS-CoV-2 infection is currently a topic of interest^12,61,62^, although not completely understood. Here we have performed detailed immunodetection studies of endogenous ACE2 and vimentin proteins, mostly in live cell culture models incubated with Spike protein constructs. As a first output, our work provides a critical assessment of multiple tools and procedures for detection of these proteins in cells. Furthermore, our results reveal a high colocalization of endogenous ACE2 with Spike, but point to a moderate overall colocalization of vimentin with Spike and ACE2 signals. Nevertheless, they highlight the presence of vimentin at selective cellular structures, namely primary cilia, where these proteins appear to be enriched. Of note, colocalization as described here indicates proximity of the proteins detected but does not imply a direct interaction, which needs to be explored by additional approaches. Altogether, the observations reported herein call for a detailed characterization of the vimentin species and their potential role in interaction with pathogens at primary cilia.

## Supplementary Information

Supplementary Information.

## Data Availability

The datasets used and/or analyzed during the current study are available from the corresponding author on reasonable request.

## Abbreviations

ACE2: Angiotensin converting enzyme 2
ARL13B: ADP-ribosylation factor-like protein 13B
CTXB: Cholera toxin B subunit
TMPRSS2: Transmembrane protease serine 2
