# Supplementary material for: Cell surface detection of vimentin, ACE2 and SARS-CoV-2 Spike proteins reveals selective colocalization at primary cilia
Source: Sci Rep. 2022 Apr 29;12:7063. doi: 10.1038/s41598-022-11248-y (PMC9052736; doi:10.1038/s41598-022-11248-y)

Cell surface detection of vimentin, ACE2 and SARS-CoV-2 Spike proteins reveals selective colocalization at primary cilia

Supplementary Information

Vasiliki Lalioti, Silvia González-Sanz, Irene Lois-Bermejo, Patricia González-Jiménez, Álvaro Viedma-Poyatos, Andrea Merino, María A. Pajares, Dolores Pérez-Sala

Department of Structural and Chemical Biology. Centro de Investigaciones Biológicas Margarita Salas, CSIC. 28040 Madrid, Spain

Short title: Cell Surface detection of vimentin, ACE2 and Spike

Please address correspondence to:

Dolores Pérez-Sala
Centro de Investigaciones Biológicas Margarita Salas, CSIC
Ramiro de Maeztu, 9
28040 Madrid, Spain
Email: dperezsala@cib.csic.es

Supplementary figures

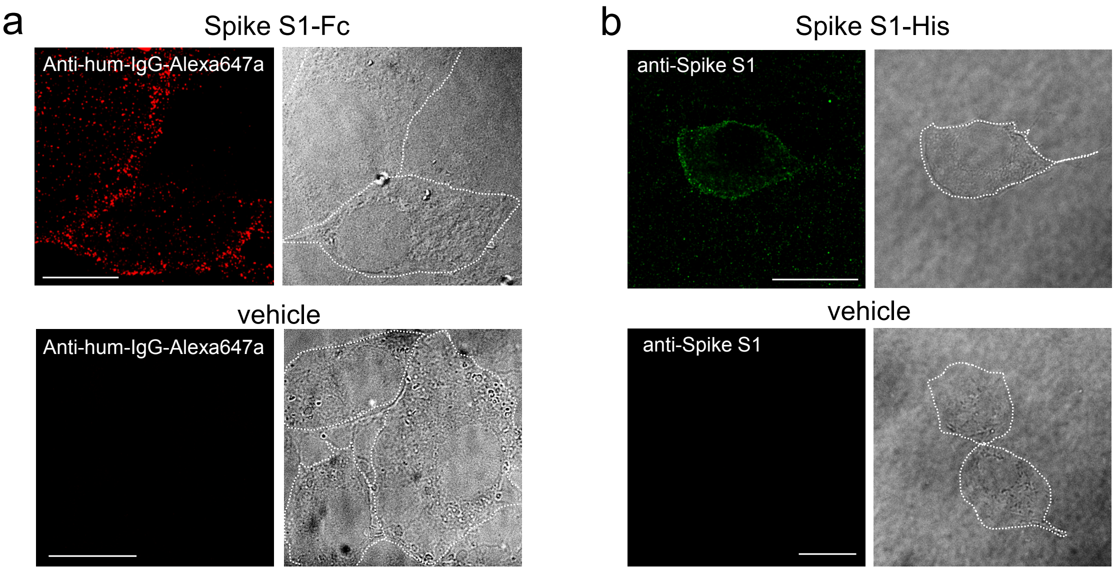

Supplementary Figure 1. Detection of Spike protein constructs at the cell surface in Vero cells. Cells plated on glass coverslips were incubated with recombinant Spike S1-Fc or vehicle, followed by detection with anti-human (anti-hum) IgG-Alexa647a (raised in goat, a) or with Spike S1-His or vehicle, followed by immunofluorescence with anti-Spike antibody (b). Single confocal sections taken at mid-height of the cells are shown at the left and bright field images in which the cell contours have been highlighted, at the right. Bars, 20 µm.

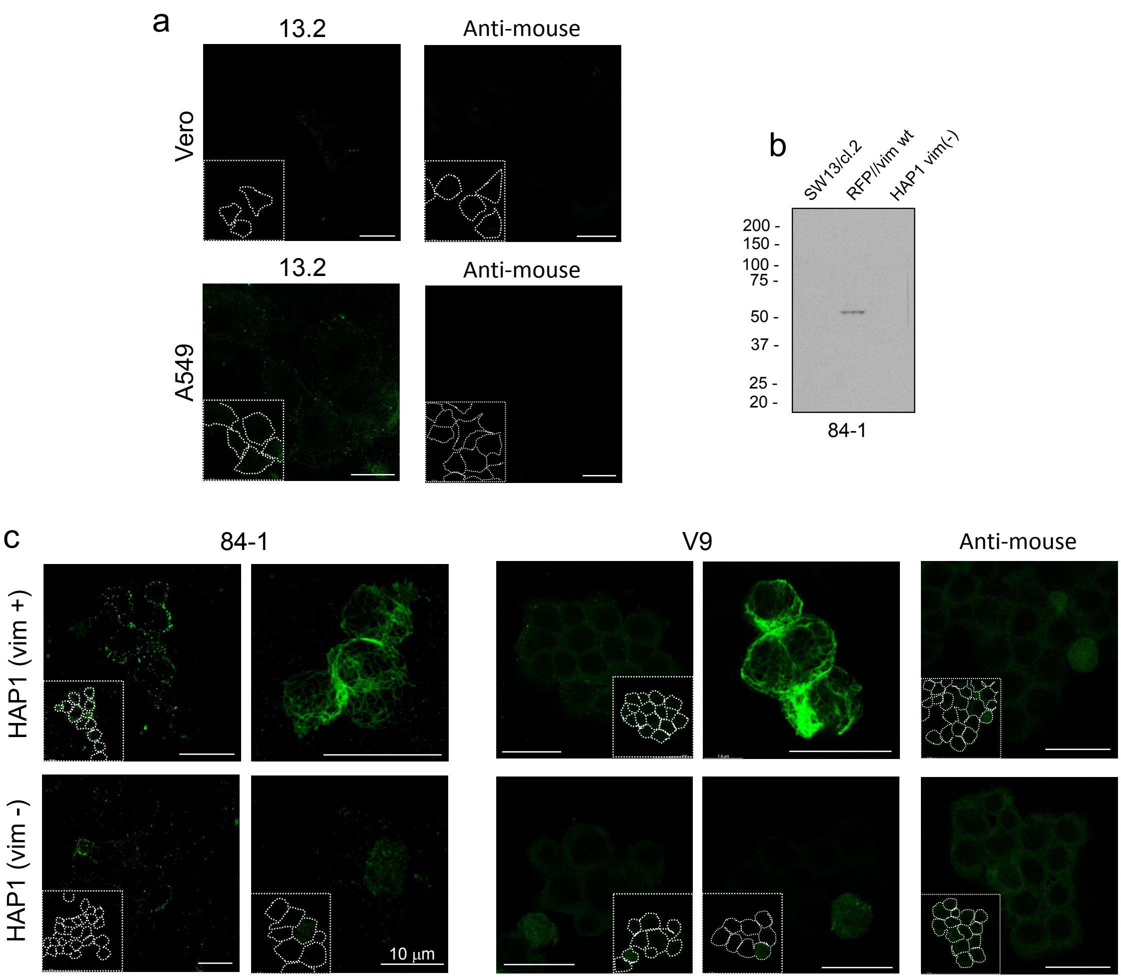

Supplementary Figure 2. Detection of vimentin in several cell types. (a) Vero or A549 cells were incubated with monoclonal antibody 13.2 at 1:200 dilution for 1 h in the cold, followed by incubation with Alexa-488-conjugated secondary antibody at 1:200 (left) or only with the secondary antibody (right). At the end of the procedure cells were fixed with 4% (w/v) PFA in the cold. (b) Immunoblot analysis of vimentin in several cell types. Total cell lysates from the indicated cell types containing 30 µg of protein were analyzed by SDS-PAGE followed by immunoblot with the 84-1 anti-vimentin antibody. SW13/cl.2 cells stably transfected with the bicistronic vector RFP//vimentin wt as detailed in Materials and Methods (RFP//vim wt) were used as positive control. (c) Vimentin-positive HAP(vim +) or vimentin-negative HAP(vim -) cells were incubated with the indicated anti-vimentin antibodies and Alexa-488-conjugated anti-mouse immunoglobulins at 1:200, before fixation (left images) or after fixation and permeabilization (right images). Insets display the approximate cell contours drawn from bright field or overexposed images. Images on the far right show the background of the secondary antibody in these cell types. Bars, 20 µm, unless otherwise indicated.

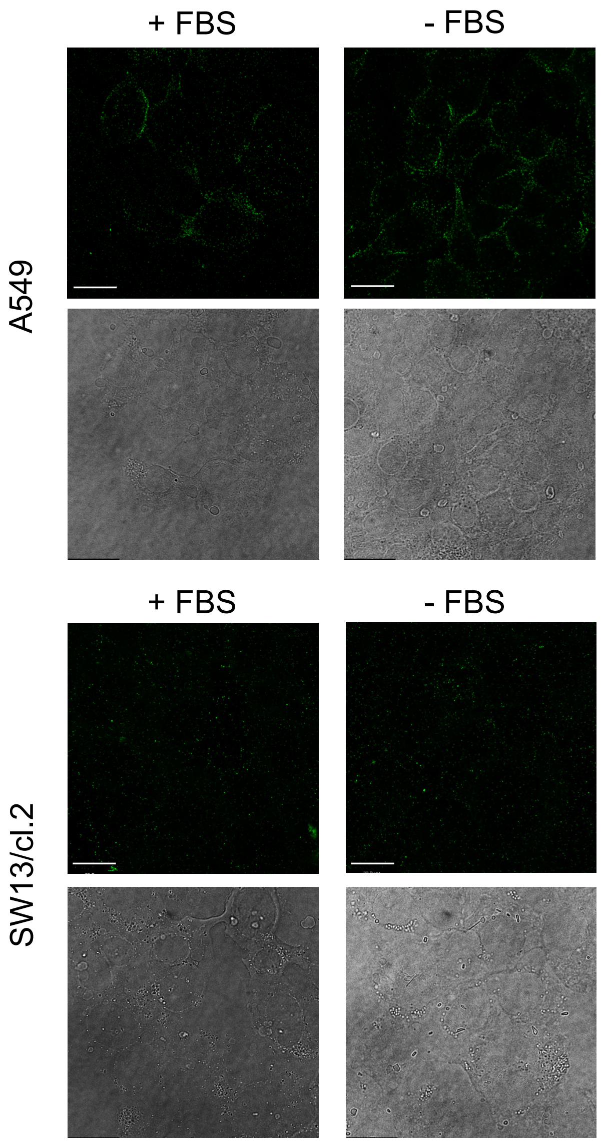

Supplementary Figure 3. Effect of serum depletion on the detection of vimentin at the cell surface. A549 or SW13/cl.2 cells were cultured in the presence or absence of FBS for 24 h before immunodetection of vimentin in live cells with the 84-1 monoclonal antibody. Images shown are fluorescent confocal single sections at mid-cell height (upper panels) and bright field images (lower panels). Bars, 20 μm.

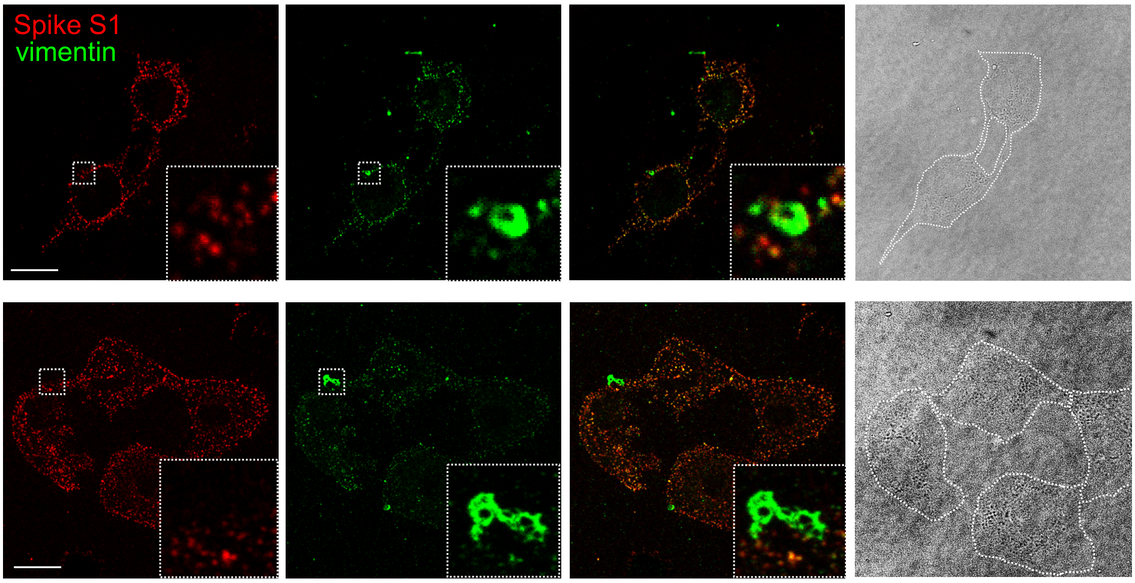

Supplementary Figure 4. Detection of Spike binding and vimentin in cells showing vimentin adhered to the cell surface. Vero cells were incubated with Spike-S1-Fc prior to immunodetection of vimentin following the protocol outlined in Figure 6 as sequence A. Single sections of the individual channels at mid cell height as well as the merged images are shown. Insets show enlarged views of the regions of interest marked by dotted squares. Panels on the right depict bright field images with the cell contours outlined. Bars, 20 µm.

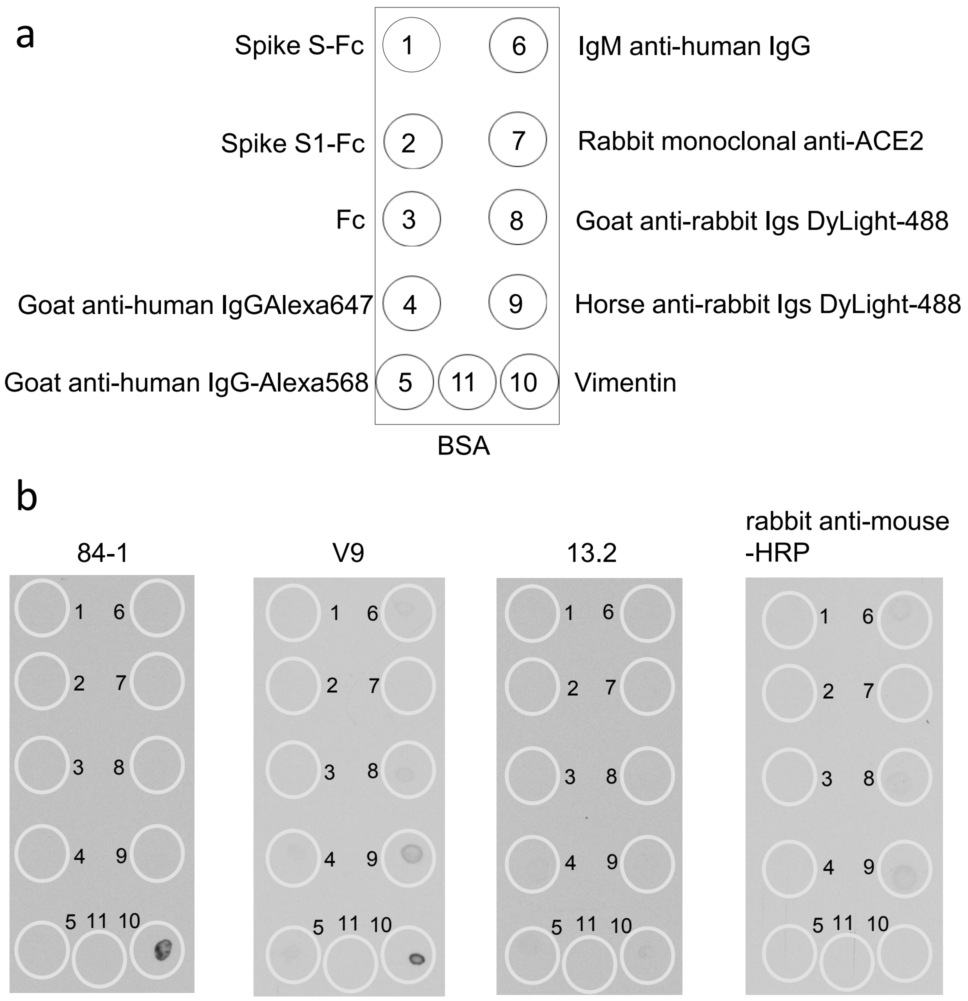

Supplementary Figure 5. Dot blot of several protein constructs and antibodies. Five μl of the indicated reagents at the concentrations used for immunodetection were spotted on nitrocellulose membrane at the positions specified in (a) and denoted by the grey circles in (b) and allowed to dry before blocking with 2% (w/v) non-fat powdered milk in T-TBS, followed by incubation with the indicated anti-vimentin antibodies at 1:500 dilution in 1% (w/v) BSA in T-TBS, and HRP-conjugated rabbit anti-mouse immunoglobulins for detection with ECL. The HRP-conjugated antibody alone was used as a control. The amounts of protein spotted in each case were as follows: Spike S-Fc, Spike S1-Fc, Fc, goat anti-human IgG-Alexa647, goat anti-human IgG-Alexa568 and BSA, 0.05 μg; IgM anti-human IgG and rabbit monoclonal anti-ACE2, 0.025 μg; goat anti-rabbit Igs DyLight-488 and horse anti-rabbit Igs DyLight-488, 0.035 μg; and vimentin 0.035 μg. Note that the 84-1 antibody only detected vimentin. The V9 antibody gave a detectable background with the anti-rabbit antibodies, spots 8 and 9, and a fainter signal with the goat anti-human IgG antibodies, spots 4 and 5. The background with anti-rabbit antibodies could be due in part to binding of the HRP-conjugated rabbit anti-mouse antibody used as secondary antibody for detection. The 13.2 antibody gave a very faint signal with recombinant vimentin. Rab bit anti-mouse-HRP gave a faint signal at spots 6, 8 and 9.

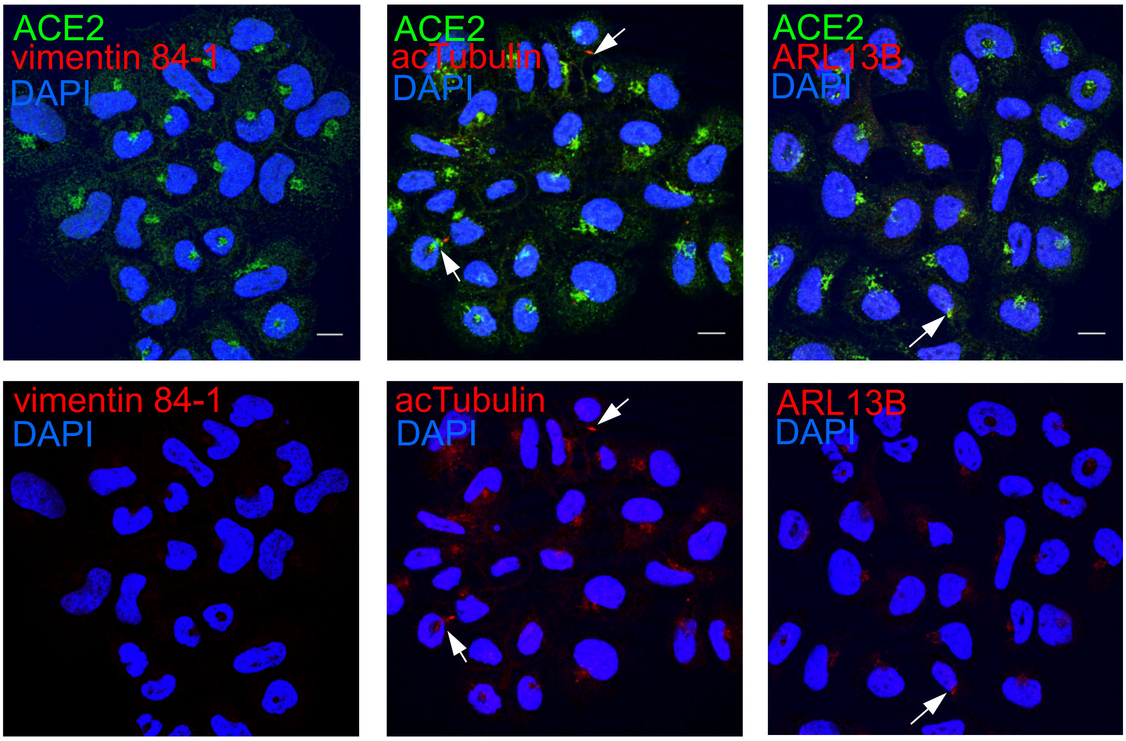

Supplementary Figure 6. Detection of vimentin and ACE2 in combination with markers of primary cilia in SW13/cl.2 cells. Vimentin-deficient SW13/cl.2 cells were fixed with 4% (w/v) PFA, permeabilized with 0.1% (v/v) Triton X-100 and stained with the indicated antibodies. Upper panels show merge images of all channels and lower panels show staining with the individual anti-vimentin, anti-acetylated tubulin or anti-ARL13B antibodies. Nuclei were counterstained with DAPI. White arrows mark incipient structures positive for acetylated tubulin or ARL13B immunoreactivity. Bars, 10 μm.

**Antibodies**							
**Antigen**	**Name**	**Type**	**Immunogen**	**Epitope**	**Vendor**	**Reference**	**Knockout validated**
**Primary antbodies**							
vimentin	V9	mouse monoclonal	purified porcine vimentin	C-terminal	Santa Cruz Biotech.	sc-6260	Yes
	13.2	mouse monoclonal	human foreskin fibroblast extract	Not determined	Sigma	V5255	
	84-1 (CSV)	mouse monoclonal	human recombinant vimentin	Putative, residues 106-113	Abnova	H00007431-M08J	
	SP20	rabbit monoclonal	human recombinant vimentin	Not determined	Thermo	MA5-14564	Yes
	C-end (vim 453-466)	goat polyclonal	peptide: QVINETSQHHDDLE	C-terminal end	Everest Biotech	EB11207	
Ace2	ACE2	rabbit polyclonal	synthetic peptide	N-terminus	Invitrogen	PA5-20045	
	ACE2 788-805	rabbit polyclonal	peptide: CKGENNPGFQNTDDVQTSF	C-terminus	Abcam	ab15348	
Spike	SARS-CoV-2 (2019-nCoV), clone 007	rabbit monoclonal		Not available	Sino Biological	40150-R007	
ARL13B	C5	mouse monoclonal		C-terminal, residues 414-428	Santa Cruz Biotech.	sc-515784	
**Secondary antibodies**							
	Anti-human IgG (H+L)-Alexa fluor 568	goat polyclonal			Invitrogen	A21090	
	Anti-human IgG Fc-Dylight 488	goat polyclonal			Invitrogen	SA5-10134	
	Anti-human IgG Alexa 647	goat polyclonal			Invitrogen	A21445	
	Anti-human IgG (H+L) Alexa fluor 647	alpaca polyclonal			Jackson Immun. Lab. Inc.	609-605-213	
	Anti-mouse F(ab')2 Alexa 488	goat polyclonal			Jackson Immun. Lab. Inc.	115-546-062	
	Anti-mouse Igs Alexa 647	goat polyclonal			Invitrogen	A21235	
	Anti-rabbit Dylight 488	horse polyclonal			Vector laboratories	DI-1088	
	Anti-rabbit Alexa 647	goat polyclonal			Invitrogen	A21244	

Supplementary Table 1. Antibodies used in this study[1].

[1]Additional technical details and discussion are available at https://www.biorxiv.org/content/10.1101/2021.05.04.442648v2.

Original blots used in figures with the cropped lanes outlined.

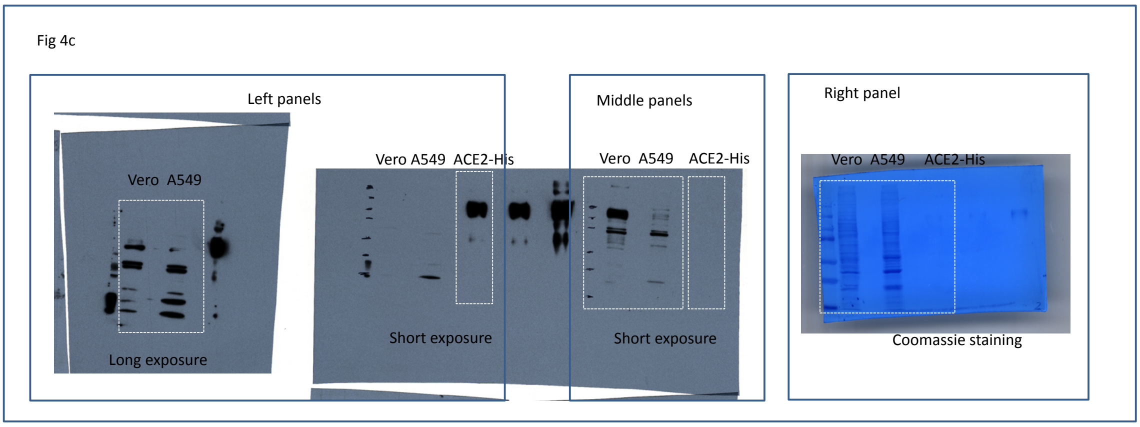

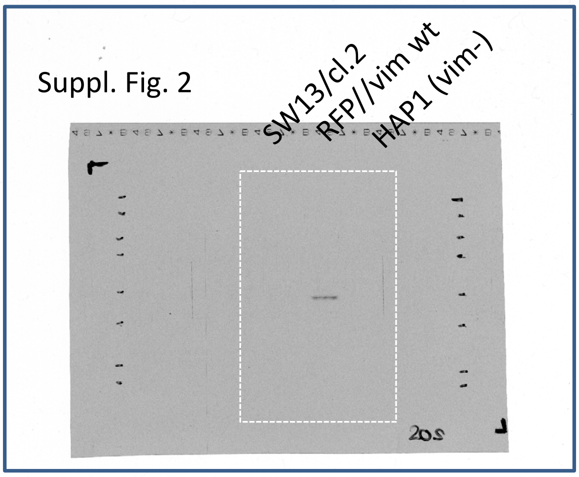

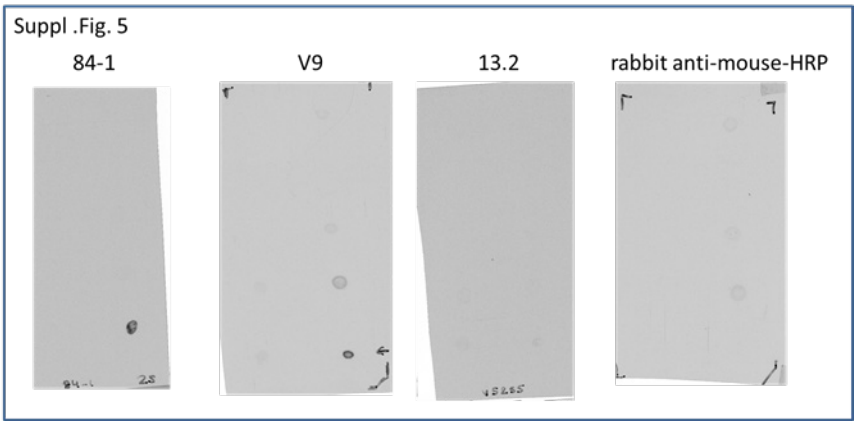

Supplement: Supplementary file 1 — Supplementary Information. [file 41598_2022_11248_MOESM1_ESM.pdf]
